# Ocean carbon sequestration during glacial climates due to combined Antarctic sea ice expansion and ocean circulation change

**DOI:** 10.1126/sciadv.ady6777

**Published:** 2026-10-21

**Authors:** Sandy Grégorio, Jonathan M. Lauderdale, Louis-Philippe Nadeau, Raffaele Ferrari

**Affiliations:** ^1^ Institut des Sciences de la Mer, Université du Québec à Rimouski, Rimouski, QC, Canada.; ^2^ Department of Earth, Atmospheric and Planetary Sciences, Massachusetts Institute of Technology, Cambridge, MA, USA.; ^3^ Center for Sustainability Science and Strategy, Massachusetts Institute of Technology, Cambridge, MA, USA.

## Abstract

Glacial-interglacial transitions punctuate Earth’s Quaternary climate, accompanied by substantial atmospheric carbon dioxide and ocean carbon storage changes. The cause of atmospheric carbon drawdown during glaciations is uncertain, involving many mechanisms whose interactions are poorly understood. Here, we show that the combined expansion of Antarctic sea ice and associated changes in the meridional overturning efficiently sequester carbon in the ocean abyss, leading to a decline in atmospheric carbon dioxide. In contrast, changes in sea ice and circulation individually result in ocean carbon dioxide outgassing. When sea ice expansion and circulation changes act together, they enhance biological carbon storage by reducing ocean-atmosphere fluxes in the Southern Ocean, without invoking external drivers of the biological pump. This reduction inhibits the outgassing of upwelled remineralized carbon and promotes its resubduction into the abyss. Together, these results reveal a reinforcing feedback linking atmospheric cooling, Antarctic sea ice expansion, and ocean circulation changes to enhanced biological carbon storage and atmospheric carbon dioxide drawdown.

## INTRODUCTION

Ice core records show that over the past 800,000 years, climate has oscillated between warm interglacial and cold glacial periods every ∼100,000 years (*1*–*3*), with glacial states characterized by 3° to 8°C cooler atmospheric temperatures (*4*–*6*), substantial Northern Hemisphere ice sheets (*7*, *8*), and expanded Antarctic sea ice (*9*, *10*). Glacial-interglacial oscillations are thought to be initiated by periodic orbital insolation forcing and amplified by positive climate feedbacks (*5*, *11*). Transitions to glacial states are marked by a substantial drop in the partial pressure of atmospheric CO_2_ (*P*co_2_^atm^) of 90 to 100 μatm (1 μatm ≡ 1 part per million at sea level), implying that *P*co_2_^atm^ changes may be an essential climate feedback (*1*–*3*).

Most of the carbon lost from the atmosphere is believed to have been sequestered in the deep ocean (*11*–*15*), but the underlying processes remain debated. Numerous mechanisms have been proposed, yet none, when considered individually, fully account for the observed atmospheric *P*co_2_^atm^ decrease (*12*, *14*). For example, increased carbon solubility in a 3°C colder ocean during the Last Glacial Maximum (LGM; ∼20,000 years ago) explains less than half of the observed drop in glacial *P*co_2_^atm^ concentrations (*11*, *12*, *14*, *16*), whereas expanded Antarctic sea ice (*10*) and changes in the large-scale ocean circulation (*17*, *18*) each lead to reduced ocean carbon storage, which, in turn, contributes to higher atmospheric *P*co_2_^atm^ (*16*, *19*, *20*). Changes in biological productivity (*11*, *13*) and ocean alkalinity (*21*) have also been invoked. Theoretical frameworks and numerical studies support the linear sum of independent mechanisms to approach the observed glacial-interglacial fluctuations in *P*co_2_^atm^ (*14*, *16*, *20*, *22*, *23*). However, whether interactions among these mechanisms amplify or counteract one another remains poorly understood and could result in substantial differences in ocean carbon storage.

In parallel to efforts to identify the drivers of *P*co_2_^atm^ drawdown, progress has been made in quantifying how ocean carbon storage is partitioned among its physical and biogeochemical components (*24*–*28*). Recent studies have focused particularly on the “disequilibrium” component, which captures the imbalance in carbon partitioning between the surface ocean and the atmosphere. This total disequilibrium can be further decomposed into physical and biological components (*16*). Khatiwala *et al.* (*16*) recently highlighted that cooler glacial temperatures reduce air-sea heat fluxes in deep-water formation regions, decreasing surface undersaturation and thereby enhancing ocean carbon storage in the physical disequilibrium component. Marzocchi and Jansen (*29*) found a substantial increase in total disequilibrium carbon storage under glacial conditions, attributed to the combined effects of expanded Antarctic sea ice and reduced mixing between the abyssal ocean and North Atlantic Deep Water (NADW). However, the differing emphasis in these studies points to an open question about the relative roles of cooling, sea ice, and circulation in driving disequilibrium.

Here, we use a suite of idealized ocean–sea ice biogeochemical model simulations to assess the individual and combined effects of physical drivers—namely, cooling, sea ice expansion, and changes in ocean circulation—on ocean carbon storage. We activate each driver alone and in combination and evaluate their impact on the different carbon components to disentangle the relative contributions of physical and biogeochemical processes to glacial carbon storage and atmospheric *P*co_2_^atm^ drawdown during glacial periods.

In our simulations, the bulk of the *P*co_2_^atm^ decline arises from a sharp increase in the disequilibrium of the biological carbon pool, triggered by Antarctic sea ice expansion and associated circulation changes, without invoking external mechanisms that increased biological productivity or efficiency (*14*, *16*). Together, these combined physical drivers reduce the exposure time of surface waters to the atmosphere in the Southern Ocean (*30*, *31*), suppressing outgassing of upwelled biologically derived carbon from the deep ocean. As a result, deep-ocean ventilation decreases, and regenerated carbon that would have been released to the atmosphere is instead resubducted into the ocean interior, leading to a sharp increase in biological carbon sequestration.

Our results show that it is the interplay between sea ice expansion and circulation changes that drives the pronounced increase in biological disequilibrium carbon storage observed in the full response. Specifically, our simulations illustrate that CO_2_ drawdown estimates based on isolated drivers miss the amplification of ocean carbon storage that arises from their combined effects. The close interplay between the physical and biological processes may have been central to sustaining the consistent pacing of glacial-interglacial CO_2_ cycles over the past 800,000 years.

## MATERIALS AND METHODS

### Modeling approach

To quantify the individual and combined effects of key ocean drivers on *P*co_2_^atm^, we run a suite of idealized model simulations forced with various combinations of glacial and interglacial ocean temperature, ocean circulation, and Antarctic sea ice concentration. We use the Massachusetts Institute of Technology general circulation model (MITgcm) (*32*), configured for the ocean with a dynamic-thermodynamic sea ice component based on viscous-plastic rheology (*33*, *34*) and an interactive biogeochemical module (*35*, *36*) that simulates the cycles of dissolved inorganic carbon (DIC), alkalinity, phosphorus, and oxygen. Preformed tracers are explicitly simulated to accurately partition total DIC into its components (*26*, *28*).

The model solves the hydrostatic Boussinesq equations on a spherical grid with a 2° horizontal resolution in both latitude and longitude and 29 vertical levels ranging from 20 m near the surface to 200 m in the deep ocean. The effects of mesoscale eddies and convection are parameterized following Gent and McWilliams (*37*) and using convective adjustment schemes, respectively. A bottom-enhanced but horizontally uniform vertical diffusivity, similar to that proposed by Nikurashin and Ferrari (*38*), is imposed to represent mixing by internal waves (fig. S1E). Momentum dissipation is represented by Laplacian viscosity, and tracer advection uses a second-order flux limiter scheme (*39*).

The idealized ocean domain geometry consists of two basins in the north connected by a circumpolar channel at the southern end (*40*, *41*), as illustrated in Fig. 1 (A and B) and fig. S1A. For clarity, we refer to the narrower basin as the Atlantic Ocean, the wider basin as the Pacific Ocean, and the circumpolar channel as the Southern Ocean.

**Fig. 1.**
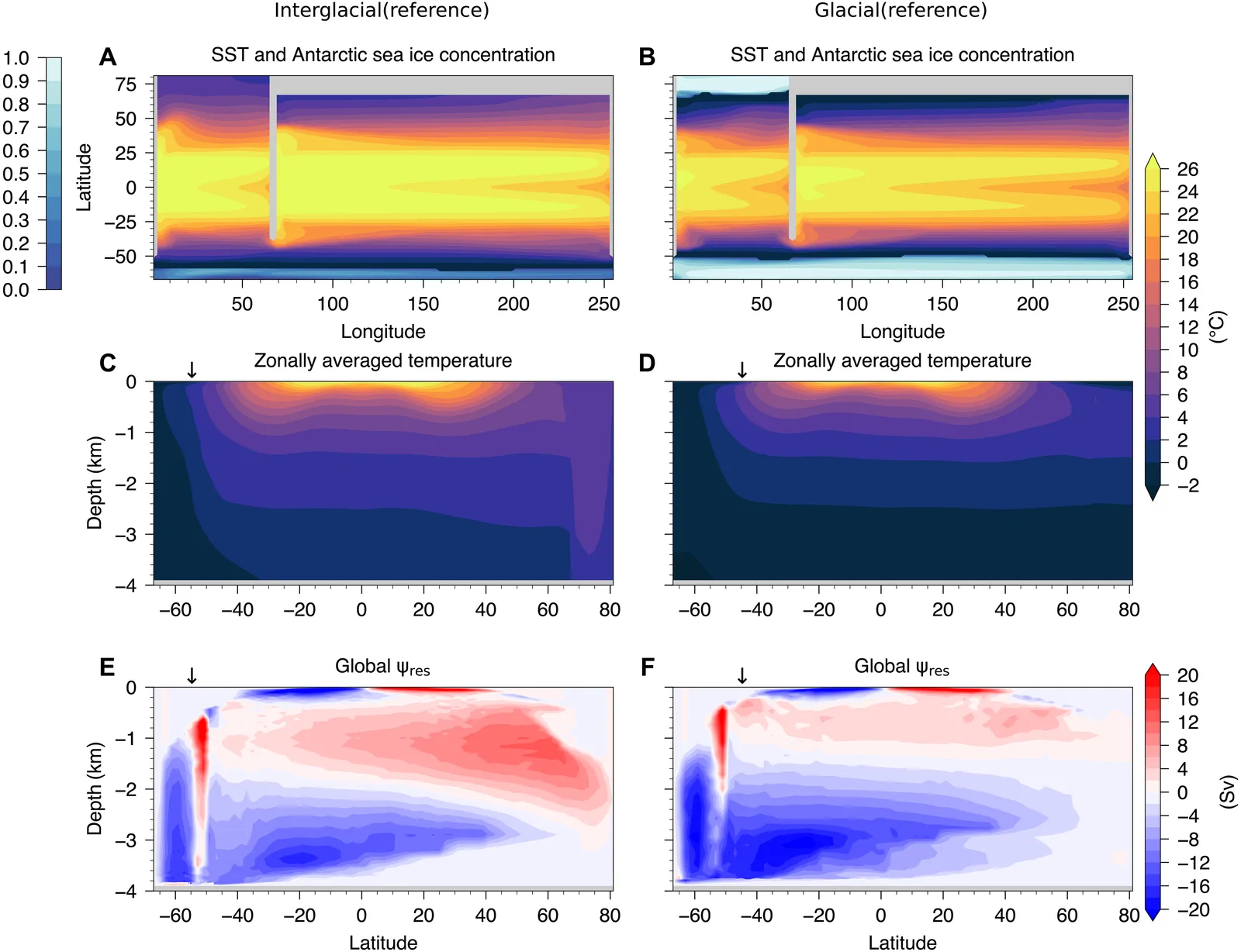
Final states of the interglacial and glacial reference simulations. Final states of Interglacial(reference) (left column) and Glacial(reference) (right column). (**A** and **B**) Sea surface temperature (SST) and sea ice concentration, (**C** and **D**) global zonally averaged temperature, and (**E** and **F**) global zonally integrated residual meridional streamfunction, ψ_res_, which combines the Eulerian-mean and eddy-induced transports (*77*). ψ_res_ is computed in density space relative to 2000 dbar and remapped to the zonally averaged depth of each density surface. Contour intervals are 2°C for temperature, 2 Sv for ψ_res_, and 0.1 for sea ice concentration [color bar to the left of (A)]. Downward black arrows (C to F) indicate the maximum zonally averaged Antarctic sea ice edge.

Surface forcing (fig. S1), including heat and freshwater fluxes, and surface wind stress are prescribed as steady, zonally averaged profiles following the approach commonly used in similar idealized modeling studies (*29*, *40*, *42*, *43*). These forcings are designed to represent long-term mean conditions rather than seasonal variability. All simulations are integrated for 10,000 years to reach statistical equilibrium, with diagnostics saved as 100-year means. The analysis is based on the final 100-year mean fields. Despite its idealized nature, this modeling framework captures key features of the large-scale ocean circulation and stratification (*40*, *41*, *44*, *45*) (see the Supplementary Materials for additional configuration details).

We refer to the simulation forced with present-day air-sea fluxes (fig. S1) as the Interglacial(reference) (Fig. 1, A, C, and E). A Glacial(reference) simulation (Fig. 1, B, D, and F) is obtained by cooling the surface restoring temperature profile used to compute the heat flux by −2°C at the equator and up to −6°C at the poles, while keeping all other forcings identical to Interglacial(reference) (fig. S1). This atmospheric cooling triggers a cascade of changes (*29*, *42*), including lower seawater temperatures, expanded Antarctic sea ice cover, and a global rearrangement of deep ocean water masses. To account for the ∼100-m eustatic drop in sea level due to accumulation of ocean water in continental ice sheets (*11*, *14*), the mean salinity in Glacial(reference) is increased by 1 psu.

Six additional simulations are designed to investigate the role of three physical drivers on ocean carbon storage: seawater temperature, sea ice, and overturning circulation. The model is configured to separately account for each of these drivers (cf. Table 1). Because both the Interglacial(reference) and Glacial(reference) simulations are integrated to equilibrium and exhibit minimal temporal variability, the last 100-year mean fields of seawater temperature and sea ice concentration from each reference scenario are used as time-invariant inputs to the biogeochemical model. To isolate the effect of temperature on a glacial climate, the model is run in the Interglacial(reference) configuration, with the biogeochemical model forced with the time-averaged temperature field from the Glacial(reference) simulation. Conversely, to isolate the effect of circulation, the model is run in the Glacial(reference) configuration, with the biogeochemical model forced with time-averaged temperature and sea ice fields from the Interglacial(reference) simulation. Simulations combining multiple drivers follow the same logic. These six simulations are run without any imposed salinity increase.

**Table 1.** Combinations of physical drivers in the idealized simulations.

Simulation	Ocean temperature	Sea ice concentration	Velocity field
Interglacial(reference)	Interglacial	Interglacial	Interglacial
Glacial(reference)	Glacial	Glacial	Glacial
Glacial(temperature)	Glacial	Interglacial	Interglacial
Glacial(sea ice)	Interglacial	Glacial	Interglacial
Glacial(circulation)	Interglacial	Interglacial	Glacial
Glacial(sea ice + temperature)	Glacial	Glacial	Interglacial
Glacial(circulation + temperature)	Glacial	Interglacial	Glacial
Glacial(sea ice + circulation)	Interglacial	Glacial	Glacial

Our analysis focuses solely on physical drivers, excluding external biogeochemical changes associated with ice sheet growth and sea level fall—such as iron fertilization and changes in ocean-wide nutrient and alkalinity concentrations—likely occurring at later stages of the glacial cycle (*14*, *46*–*50*). Furthermore, the model represents a closed ocean-atmosphere carbon system and does not include terrestrial carbon reservoirs, riverine inputs, sedimentary interactions, or weathering. Hence, we do not expect to reproduce the full 90-μatm glacial CO_2_ drawdown recorded in proxy data.

### Carbon framework

Carbon storage is evaluated by partitioning DIC into components reflecting the effects of different processes (*16*, *24*–*26*, *28*, *29*). DIC is first decomposed into two pools: preformed carbon, *C*_pre_, and regenerated carbon, *C*_reg_. *C*_pre_ represents the DIC content of a surface water parcel at the time of subduction, whereas *C*_reg_ corresponds to the DIC accumulated in the ocean interior through the remineralization of particulate organic and inorganic carbon after subduction (*24*, *25*).

*C*_pre_ is further divided into solubility, *C*_sol_, and disequilibrium, *C*_dis_, components. *C*_sol_ represents the DIC expected if surface waters were in equilibrium with the atmosphere and depends primarily on local seawater temperature, salinity, and alkalinity. *C*_sol_ is computed from the 100-year mean fields of potential temperature, θ, salinity, *S*, and preformed alkalinity, *A*_pre_, together with the prognostic atmospheric *P*co_2_^atm^ of each scenario. Calculations are performed using the PyCO2SYS Python toolbox (*51*, *52*), which solves the full nonlinear carbonate system.

The disequilibrium component, defined as $C_{\text{dis}}=C_{\text{pre}}-C_{\text{sol}}$, accounts for oceanic carbon deficit or excess due to physical and biological processes that drive surface waters away from the equilibrium concentration. We conduct a set of simulations with the biological activity switched off to estimate the physical disequilibrium reservoir, *C*_disP_. This approach isolates the effect of cooling waters that begin to absorb CO_2_ and are subducted into the ocean interior before fully equilibrating with the atmosphere. The biological disequilibrium reservoir, *C*_disB_, is then calculated as the residual between *C*_dis_ and *C*_disP_ to isolate the effect of trapping upwelled regenerated carbon that is only partially outgassed before being subducted into the ocean interior, particularly in the Southern Ocean (*25*, *26*, *28*, *30*).

We use explicitly simulated preformed tracers to diagnose the carbon reservoirs rather than AOU-based estimates, which tend to overestimate regenerated carbon and underestimate disequilibrium in high-latitude regions where gas exchange is limited (*16*, *53*) (see the Supplementary Materials). We then compute changes in each carbon reservoir in response to the various “glacial perturbations,” compared to the interglacial state.

Changes in *C*_sol_ are partitioned into physically and biogeochemically driven components. Changes in the physical solubility pool, $\Delta C_{\text{sol}}\left(\theta ,S\right)∼\Delta C_{\text{sol}}\left(\theta \right)+\Delta C_{\text{sol}}\left(S\right)$, are associated with changes in seawater temperature and salinity. Changes in the biological solubility pool, $\Delta C_{\text{sol}}\left(A_{\text{pre}}\right)$, are related to surface changes in preformed alkalinity driven by calcium carbonate formation and uptake of charged nutrients (*29*). Each contribution is evaluated by recomputing *C*_sol_ while perturbing one variable at a time between the interglacial and glacial fields. For instance, $\Delta C_{\text{sol}}\left(\theta \right)=C_{\text{sol}}\left(\theta^{G},S^{I},A_{T}^{I},P{\text{CO}}_{2}{}^{{\mathrm{atm}}^{I}}\right)-C_{\text{sol}}\left(\theta^{I},S^{I},A_{T}^{I},P{\text{CO}}_{2}{}^{{\mathrm{atm}}^{I}}\right)$, where G and I stand for glacial and interglacial, respectively. Analogous definitions apply for *S* and *A*_T_. This “one-at-a-time” approach isolates the individual effect of each driver on Δ*C*_sol_, while effectively providing a local linearization of the carbonate system around equilibrium conditions, consistent with the framework of Lauderdale *et al.* (*27*). Further details on the carbon budget decomposition are provided in the Supplementary Materials.

Thus, changes in DIC can be broadly attributed to physical, $\Delta C_{\text{phys}}$, and biogeochemical, $\Delta C_{\text{bgc}}$, contributions

$$\begin{array}{c} \Delta \text{DIC}=\underset{\Delta C\text{phys}}{\underbrace{\Delta C_{\text{sol}}\left(\theta \right)+\Delta C_{\text{sol}}\left(S\right)+\Delta C_{\text{disP}}}}\\ +\underset{\Delta C_{\text{bgc}}}{\underbrace{\Delta C_{\text{sol}}\left(A_{\text{pre}}\right)+\Delta C_{\text{reg}}+\Delta C_{\text{disB}}}} \end{array}$$ (1)

with the separate detailed components providing further insight.

Central to interpreting the results below, the integrated biological disequilibrium, *C*_disB_, in the Interglacial(reference) simulation is always positive, reflecting the incomplete outgassing of upwelled regenerated carbon and thus indicative of carbon sequestration (*26*, *28*, *30*) (fig. S2, C and D). In contrast, the physical disequilibrium, *C*_disP_, is negative, resulting from the incomplete uptake of carbon by surface waters that have cooled and begun to sink (fig. S2, A and B). Thus, a positive Δ*C*_disB_ anomaly indicates increased ocean-atmosphere disequilibrium (i.e., oversaturation) and enhanced biological carbon sequestration. In contrast, a positive Δ*C*_disP_ anomaly reflects a decrease in physical disequilibrium (i.e., reduced undersaturation) due to surface waters closer to equilibrium with the atmosphere.

## RESULTS

### From interglacial to glacial climate

#### Physical features

Figure 1, fig. S3, and Table 2 summarize the main physical characteristics of the interglacial and glacial reference simulations. Each simulation captures key features of the interglacial and glacial ocean states, falling within the range of observational and proxy estimates (Table 2).

**Table 2.** Physical features of the reference simulations. Anomalies are glacial relative to interglacial values. 1 Sverdrup (Sv) ≡ 10^6^ m^3^ s^−1^. References are listed alongside observational and proxy estimates. Antarctic sea ice ranges for the observational and proxy estimates correspond to summer-winter estimates. The observational/proxy estimate of the mean interglacial Antarctic sea ice edge latitude uses an inferred winter value, deduced from the glacial-interglacial latitudinal shift reported by Gersonde *et al.* (*9*), combined with the glacial winter ice edge estimate from Green *et al.* (*78*).

Metric	Units	Model	Observation/proxy
Interglacial mean ocean temperature	°C	+4.1	+3.8 (*79*)
Mean ocean temperature anomalies	°C	−2.0	−2.6 ± 0.2 (*80*)
Interglacial sea surface temperature	°C	+18.3	+18.3 ± 0.5 (*79*)
Sea surface temperature anomalies	°C	−3.1	−1.9 ± 1.8 (*81*)
Interglacial Antarctic sea ice area	10^6^ km^2^	3.5	3.1–18.5 (*82*)
Glacial Antarctic sea ice area	10^6^ km^2^	21.5	14.5 ± 0.5–35.5 ± 0.5 (*78*)
Zonal mean interglacial Antarctic sea ice edge latitude	°S	59	61.0 ± 0.5–56.0 ± 0.5 (*9*, *78*)
Zonal mean glacial Antarctic sea ice edge latitude	°S	49	61.5 ± 0.5–51 ± 0.5 (*78*)
Interglacial upper cell overturning transport	Sv	+13.3	+17.2 ± 3.3 (*83*)
Glacial upper cell overturning transport	Sv	+4.5	–
Interglacial lower cell overturning transport	Sv	−17.6	−20.9 ± 6.7 (*83*)
Glacial lower cell overturning transport	Sv	−21.4	–

The global overturning circulation in the interglacial simulation exhibits the familiar two-cell structure, with a mid-depth cell representing the Atlantic Meridional Overturning Circulation (AMOC) fueled by NADW formation and an abyssal cell associated with Antarctic Bottom Water (AABW) formation (Fig. 1E). The apparent two-cell pattern arises from zonal averaging, which compresses the looping three-dimensional circulation into a two-dimensional plane (*54*).

Cooling the global climate results in a pronounced shoaling and weakening of the AMOC (*16*, *55*), accompanied by a thickening and strengthening of the abyssal cell. The latitude of AABW upwelling shifts northward from 61°S to about 53°S, consistent with the 10° expansion of Antarctic sea ice.

#### Interglacial ocean carbon budget

The distribution of carbon and nutrients in Interglacial(reference) aligns well with present-day observations (*56*). At global scale, the bulk of DIC concentration resides in the solubility pool (table S1). DIC has a pronounced vertical gradient reflecting the depletion of upper ocean nutrient concentrations (figs. S4 and S5) due to export production and the remineralization of sinking particles, defining the biological pump, *C*_reg_ (*24*). Negative physical disequilibrium concentrations (fig. S2, A and B, and table S1) indicate incomplete carbon uptake from the atmosphere, while positive biological disequilibrium (fig. S2, C and D) indicates incomplete outgassing of upwelled regenerated carbon set during Southern Ocean deep water mass formation, consistent with other modeling studies (*16*).

#### Fingerprint of the glacial ocean carbon

Figure 2 presents anomalies in ocean carbon storage, decomposed into physical, Δ*C*_phys_, and biogeochemical, Δ*C*_bgc_, reservoirs (Eq. 1) for the whole set of simulations, relative to Interglacial(reference) (sections shown in figs. S6 and S7). The transition from interglacial to glacial states, with all drivers operating concurrently, results in a *P*co_2_^atm^ drawdown of 42 μatm, predominantly driven by biogeochemically sequestered carbon (Δ*C*_bgc_ > 80%; Fig. 2A). A comparison of physical and biogeochemical contributions to *P*co_2_^atm^ anomalies across the “individual” and “combined” driver simulations reveals that only Glacial(sea ice + circulation) reproduces Δ*C*_bgc_ levels comparable to Glacial(reference) (Fig. 2, A and B). Temperature alone achieves substantial CO_2_ drawdown, primarily attributable to Δ*C*_phys_, as will be discussed in a subsequent subsection (Fig. 2E).

**Fig. 2.**
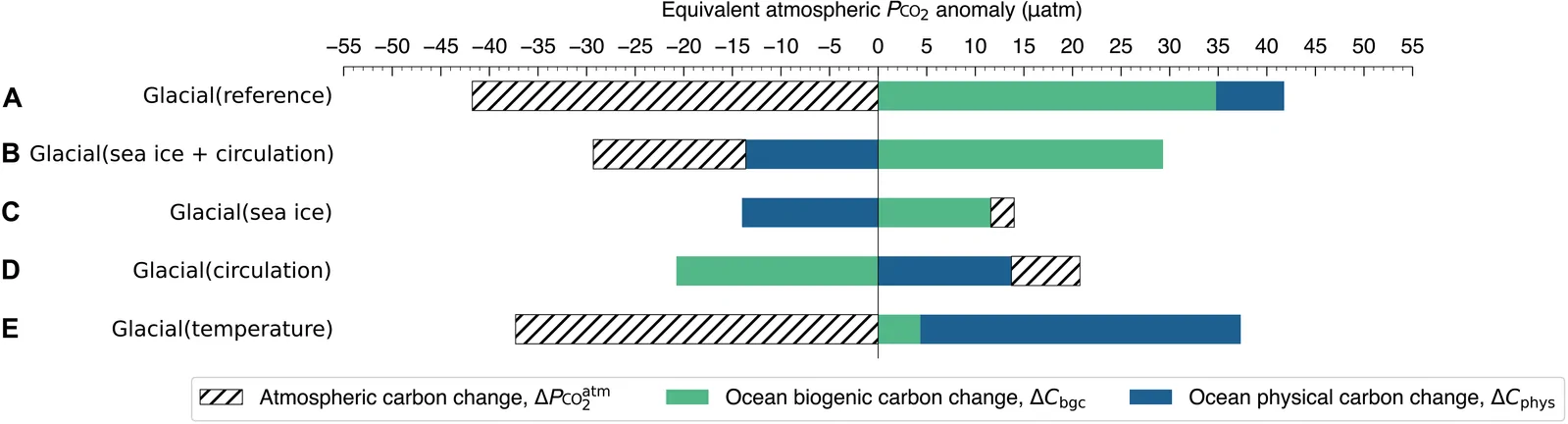
Partitioning of atmospheric and oceanic carbon anomalies in response to glacial perturbations. Atmospheric (white boxes with hatching) and oceanic (green and blue) carbon pool anomalies for (**A**) Glacial(reference), (**B**) Glacial(sea ice + circulation), (**C**) Glacial(sea ice), (**D**) Glacial(circulation), and (**E**) Glacial(temperature), relative to Interglacial(reference). The oceanic carbon reservoir is divided into biogeochemical (*C*_bgc_, green) and physical (*C*_phys_, blue) components, with $\Delta C_{\text{bgc}}=\Delta C_{\text{sol}}\left(A_{\text{pre}}\right)+\Delta C_{\text{disB}}+\Delta C_{\text{reg}}$ and $\Delta C_{\text{phys}}=\Delta C_{\text{sol}}\left(\theta \right)+\Delta C_{\text{sol}}\left(S\right)+\Delta C_{\text{disP}}$, as computed from the individual anomaly terms listed in table S2. These pools are expressed in μatm to allow direct comparison of their contributions to *P*co_2_^atm^ changes (see the Supplementary Materials for details).

Figure 3 further breaks down the carbon anomalies into the components defined in Eq. 1, allowing us to identify the contribution of each reservoir. The gray hexagon in the background of each panel represents zero anomalies, while the overlaid colored shape shows anomalies in the carbon reservoirs. Carbon pools associated with Δ*C*_phys_ and Δ*C*_bgc_ form, respectively, the top and bottom of the hexagon. Values outside the gray hexagon indicate ocean carbon uptake, whereas those inside represent ocean carbon loss. Thus, the colored shapes provide a visual “fingerprint” of carbon budget changes relative to Interglacial(reference). Figure 3 confirms that, among all individual and combined driver simulations, only Glacial(sea ice + circulation) exhibits a nearly identical fingerprint to Glacial(reference). This strong similarity suggests that the combined effects of Antarctic sea ice expansion and ocean circulation changes play a dominant role in shaping the glacial carbon budget. The substantial increase in Δ*C*_bgc_ is primarily driven by carbon accumulation in the biological disequilibrium reservoir, Δ*C*_disB_, while both simulations also exhibit a marked decline in the regenerated pool, Δ*C*_reg_ (Fig. 3, A and B). The next section explores in greater detail how Antarctic sea ice expansion and ocean circulation changes interact to drive the sharp increase in carbon storage within the biological disequilibrium pool, Δ*C*_disB_.

**Fig. 3.**
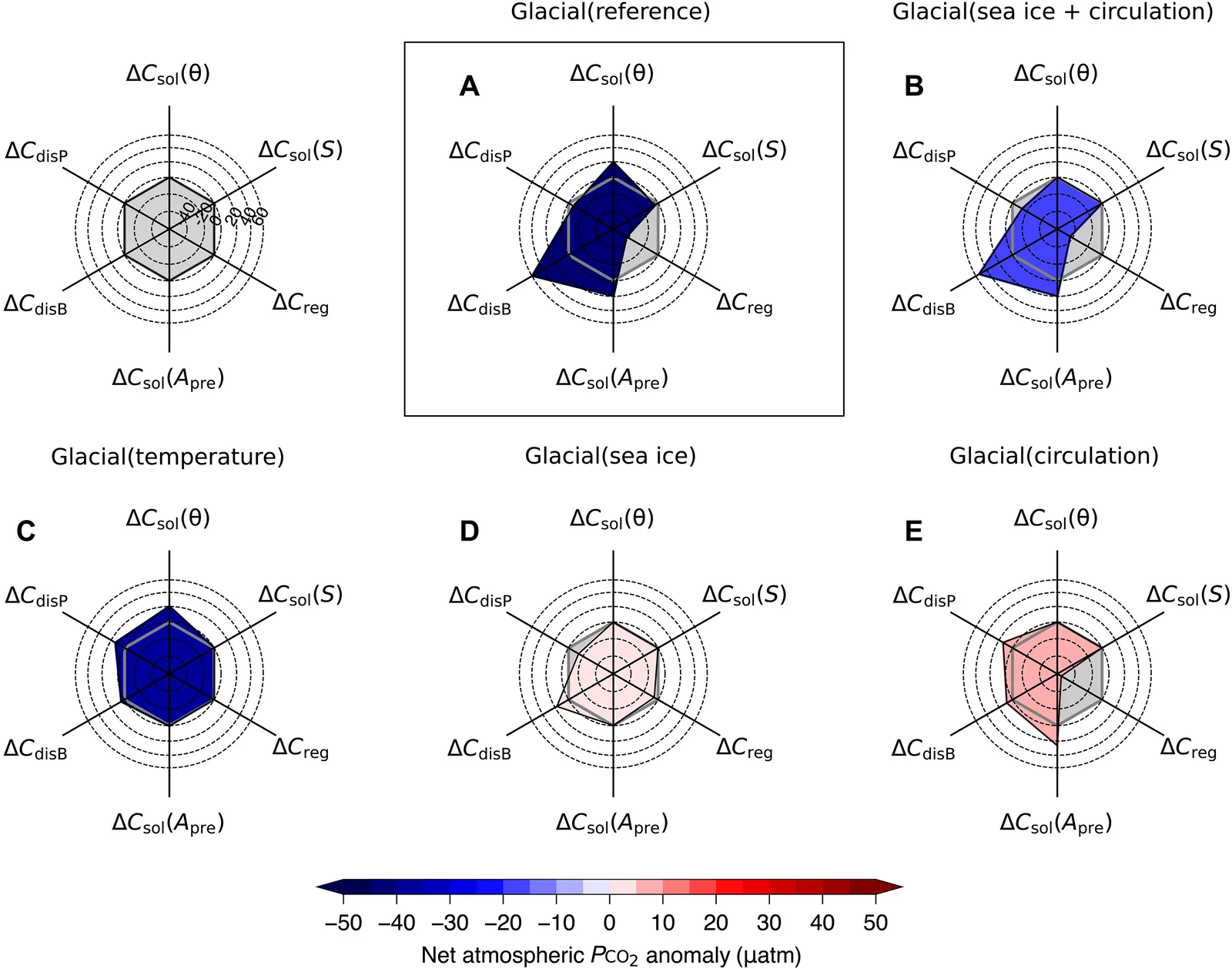
Detailed partitioning of oceanic carbon anomalies in response to glacial perturbations. Detailed oceanic carbon pool anomalies (μatm) for (**A**) Glacial(reference), (**B**) Glacial(sea ice + circulation), and (**C** to **E**) individual driver experiments relative to Interglacial(reference). Gray hexagons denote zero anomalies, with colored areas outside the gray perimeter indicating ocean carbon uptake and those inside indicating ocean carbon loss. Dashed circles are 20-μatm intervals. All anomaly values used to construct the figure are taken from the individual components reported in table S2. Color shows Δ*P*co_2_^atm^ (in μatm) relative to Interglacial(reference).

### Sea ice expansion and circulation change: Amplified biological carbon storage due to interacting physical drivers

We first examine sea ice expansion and circulation changes in isolation to better understand their separate effects on carbon storage before turning to their combined impact. Individually, these mechanisms produce compensating and opposing responses in Δ*C*_phys_ and Δ*C*_bgc_, both resulting in a net small ocean CO_2_ outgassing (Fig. 2, C and D).

The expansion of Antarctic sea ice alone, as in Glacial(sea ice), reduces contact with the atmosphere in the Southern Ocean’s upwelling region. This inhibits CO_2_ exchange, which manifests as opposing effects on the biological and physical disequilibrium reservoirs (Fig. 3D and fig. S7, A and B). Thicker sea ice and its equatorward expansion (Fig. 1B) suppress outgassing of upwelled regenerated carbon in the southward-flowing branch of the Southern Ocean overturning, increasing Δ*C*_disB_. At the same time, reduced air-sea contact limits solubility-driven CO_2_ uptake in regions of ocean heat loss (*19*, *57*, *58*), making the ocean more undersaturated, that is, leading to a more negative physical disequilibrium.

When circulation changes occur in isolation, as in Glacial(circulation), the reorganization of water masses (Fig. 1F and fig. S3, B and D) (*59*) leads to a deeper nutricline (fig. S5). This results from the shoaling of nutrient-poor NADW above ∼2000 m into a region of weak vertical mixing, reducing the exchange of tracers between the mid-depth and abyssal overturning cells (*40*, *60*) and effectively isolating nutrient-rich deep waters from the upper ocean. Consequently, the nutrient supply from Southern Ocean–sourced waters to the thermocline is weakened (*61*–*63*), culminating in a 35% reduction in biological productivity and a substantial decline in regenerated carbon storage, Δ*C*_reg_ (Fig. 3E and fig. S6D). This sharp carbon loss is, however, nearly offset by a compensating gain in other reservoirs (Fig. 3E and fig. S7, C and D).

The details of this offset are quite complex, and we discuss them here for completeness. Reduced biological productivity weakens the export of particulate inorganic carbon (carbonate) to depth, raising surface alkalinity and enhancing carbon uptake in the solubility reservoir, Δ*C*_sol_(*A*_pre_). In parallel, changes in water mass distributions also account for carbon increases in biological and physical disequilibrium reservoirs. In Interglacial(reference), NADW is more undersaturated than AABW (more negative *C*_disP_; fig. S2A) because it forms from warmer, saltier waters that convect before fully equilibrating with atmospheric CO_2_, unlike the cooler Southern Ocean waters that form AABW. In contrast, AABW carries higher biological disequilibrium carbon than NADW (fig. S2, C and D) because upwelled, regenerated carbon-rich waters flowing southward are rapidly resubducted into the abyssal cell, leaving insufficient time for full air-sea equilibration. Under glacial circulation, the contraction of relatively low-*C*_disB_, more undersaturated *C*_disP_ NADW and the expansion of relatively high-*C*_disB_, less undersaturated *C*_disP_ AABW lead to carbon increases in both Δ*C*_disB_ (more positive *C*_disB_; Fig. 3E and fig. S7D) and Δ*C*_disP_ (less negative *C*_disP_; Fig. 3E and fig. S7C). Overall, the glacial circulation scenario leads to a net carbon loss in the regenerated reservoir, partly balanced by a carbon gain in the solubility and in the biological and physical disequilibrium reservoirs, consistent with the results of Khatiwala *et al.* (*16*).

Individually, sea ice expansion and circulation changes result in a moderate increase in Δ*C*_disB_ (Fig. 3, D and E, and fig. S7, B and D). However, when combined, these two drivers amplify carbon storage in Δ*C*_disB_ (Fig. 3B and fig. S7F). To better understand this sharp increase, we revisit the definition of *C*_disB_ and the processes that govern its accumulation in the deep ocean.

The biological disequilibrium pool, *C*_disB_, is set during subduction and subsequently behaves as a passive tracer in the ocean interior. This contrasts with the regenerated carbon pool, *C*_reg_, which accumulates progressively in the subsurface by remineralization of sinking organic matter. As a result, *C*_reg_ is typically close to zero at the point of subduction, while *C*_disB_ enters the ocean interior following water mass transformation and circulation. The global distribution of *C*_disB_ is therefore primarily controlled by surface conditions in deep water formation regions (*30*).

In upwelling regions such as the Southern Ocean, deep waters rich in *C*_reg_ reach the surface supersaturated and release excess carbon to the atmosphere. However, not all of this carbon is outgassed before the waters are resubducted. The fraction that remains forms *C*_disB_. The fate of this biological disequilibrium depends on the pathways taken by upwelled waters. As deep water upwells in the Southern Ocean, part of it flows equatorward within the upper overturning cell, while another portion moves southward and is transformed into AABW (*30*). Since AABW-forming waters are subducted considerably more rapidly than those in the upper cell, they have much less time to equilibrate with the atmosphere and thus retain a larger fraction of *C*_disB_, setting the biological disequilibrium stored in the deep ocean (*30*).

#### The role of air-sea equilibration time in the Southern Ocean

Ito and Follows (*30*) developed a theoretical framework predicting that the accumulation of biological disequilibrium carbon in the ocean interior depends on the efficiency with which air-sea gas exchange erodes disequilibrium, while surface waters remain exposed to the atmosphere in the Southern Ocean. They showed that this efficiency scales with the nondimensional ratio τ_res/_τ_gas_, where τ_res_ is the residence time of surface waters between upwelling and resubduction in the Southern Ocean [see also (*31*)] and τ_gas_ is the characteristic gas equilibration timescale for CO_2_. Biological disequilibrium is also affected by the continuous upwelling of regenerated carbon into the surface mixed layer. We first discuss the result of Ito and Follows (*30*), which assumes that there is no upwelling of regenerated carbon as waters flow southward in the mixed layer, but then illustrate that the impact of this upwelling is in fact leading order.

Following Ito and Follows (*30*), we diagnose a characteristic Southern Ocean surface residence time in our simulations and examine the relationship between τ_res/_τ_gas_ and the fraction of biological carbon in upwelling waters that is retained as disequilibrium carbon upon subduction, $\frac{C_{\text{disB}}}{C_{\text{disB}}+C_{\text{reg}}}$, where *C*_disB_ and *C*_reg_ are computed as Southern Ocean averages (Fig. 4). *C*_disB_ appears also in the denominator because the biological carbon concentration of waters upwelling into the Southern Ocean surface mixed layer is given by the sum of the disequilibrium carbon previously subducted and conservatively transported through the ocean interior plus the regenerated carbon accumulated along the abyssal circulation pathway.

**Fig. 4.**
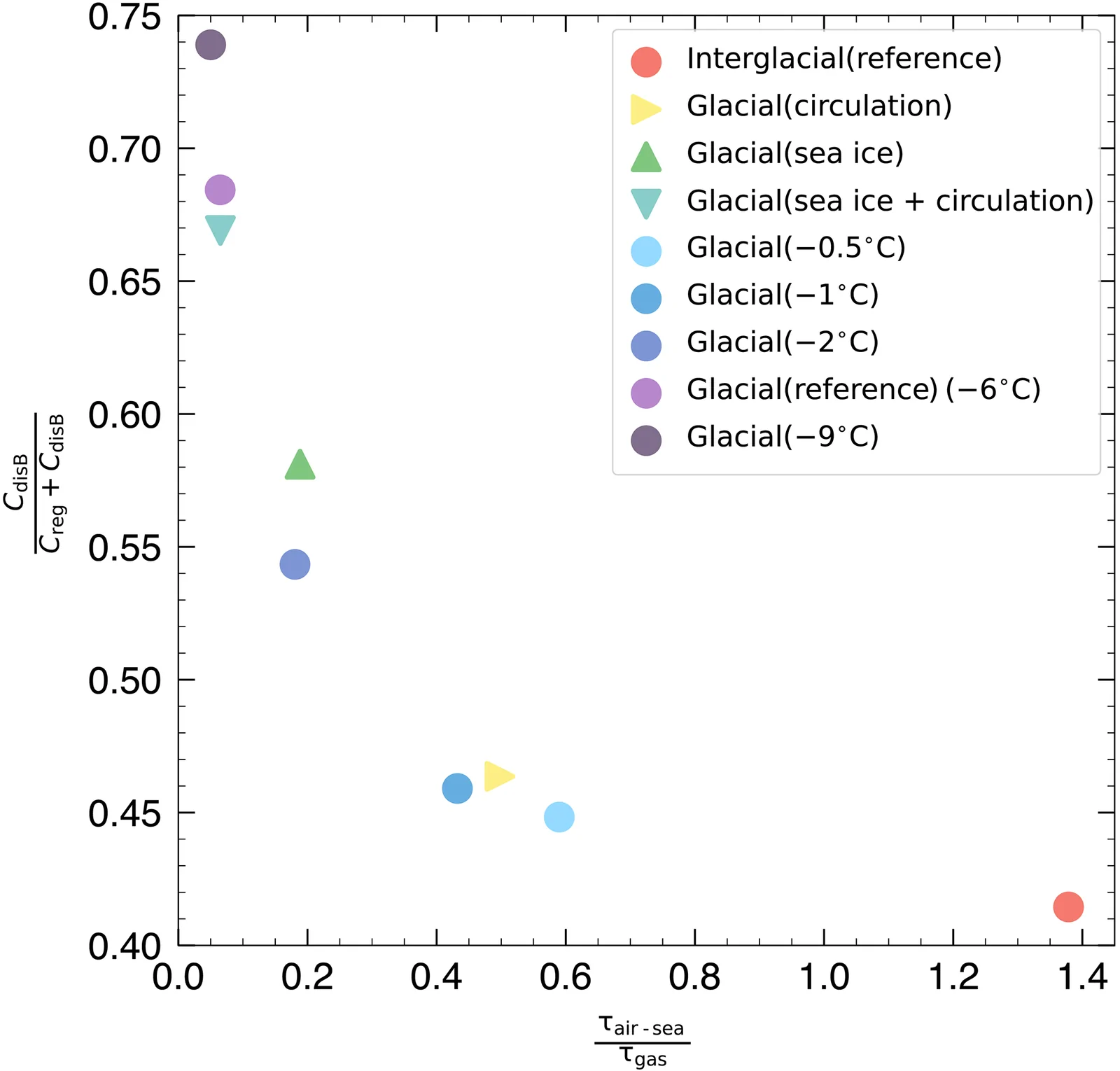
Dependence of biological disequilibrium carbon storage on Southern Ocean air-sea equilibration time. Southern Ocean–averaged fraction of biological carbon stored as disequilibrium carbon, *C*_disB_/(*C*_disB_ + *C*_reg_), as a function of the ratio τ_air-sea_/τ_gas_, where τ_air-sea_ is the Southern Ocean air-sea equilibration time and τ_gas_ is the CO_2_ gas equilibration timescale. The disequilibrium fraction is averaged below the mixed layer between the southernmost outcropping isopycnal and the isopycnal separating the upper and lower overturning cells at the end of the Southern Ocean channel. Values of τ_air-sea_ and τ_gas_ are given in table S3.

The residence time is defined as $\tau_{\text{res}}=\ell /v_{\text{res}}$, where ℓ is the latitudinal extent of the southward-flowing surface branch between the upwelling and subduction regions and $v_{\text{res}}$ is the residual meridional velocity. The latter is given at the latitude *y* by $\;v_{\text{res}}\left(y\right)=\psi_{\text{res}}\left(y,h\right)/(Lh)$, where *L* is the zonal extent of the Southern Ocean, *h* is the zonally averaged mixed layer depth, and $\psi_{\text{res}}\left(y,h\right)$ is the residual streamfunction computed at the base of the mixed layer. However, the presence of sea ice limits the area available for gas exchange, effectively reducing the time available for equilibration with the atmosphere. To account for this, we define the air-sea equilibration window, $\tau_{\text{air}‐\text{sea}}=\left(1-c\right)\ell /v_{\text{res}}$, where *c* is the fractional sea ice cover, such that only the ice-free fraction of the trajectory contributes to gas exchange (*31*). By definition, τ_air-sea_ may be shorter than the physical residence time τ_res_ that a water mass spends in the surface layer. We diagnose τ_air-sea_ from the model by integrating $\left[1-c\left(y\right)\right]\mathrm{Lh}\left(y\right)/\psi_{\text{res}}\left(y,h\right)$ over all channel latitudes along ℓ. The gas equilibration timescale, τ_gas_, which depends on the mixed layer depth, wind speed, and temperature-dependent Schmidt number (*64*), is diagnosed over the same Southern Ocean latitudinal range.

Across all simulations, the fraction of biological carbon stored as disequilibrium carbon, *C*_disB_/(*C*_disB_ + *C*_reg_), exhibits a pronounced increase as τ_air-sea_/τ_gas_ decreases (Fig. 4), particularly below the threshold where τ_air-sea_ = τ_gas_. In Interglacial(reference) where τ_air-sea_ > τ_gas_, less than 40% of the biological carbon transported into the Southern Ocean surface layer by upwelling is retained as disequilibrium carbon, whereas this fraction increases to more than 65% in Glacial(reference), where τ_air-sea _ << τ_gas_. Thus, shorter air-sea exchange times relative to the gas equilibration timescale lead to a more efficient trapping of upwelled biological carbon as *C*_disB_.

We performed four additional glacial simulations, three restored to warmer and one to a colder sea surface temperature than in Glacial(reference) (fig. S1B; the corresponding carbon budgets are detailed in figs. S8 and S9). Across the full set of experiments, including simulations in which sea ice and circulation are modified individually or in combination, decreasing τ_air-sea_/τ_gas_ consistently increases disequilibrium carbon storage.

The results of Fig. 4 support the framework of Ito and Follows (*30*) in that shorter air-sea equilibration times relative to the gas equilibration timescale favor the retention of biological carbon as disequilibrium carbon. However, the simulations deviate from the simple exponential behavior predicted by Ito and Follows (*30*), especially at large τ_air-sea_/τ_gas_, where τ_air-sea_ > τ_gas_ would imply more extensive equilibration. This demonstrates that additional processes contribute to sustaining a finite fraction of disequilibrium carbon prior to subduction.

A likely explanation is that biological carbon upwells over a broad area of the Southern Ocean, continuously supplying the surface waters with regenerated carbon, as illustrated in Fig. 5 (A and B) for both Interglacial(reference) and Glacial(reference) experiments. Despite the loss of disequilibrium through air-sea fluxes, *C*_disB_ increases as waters flow poleward, particularly beneath the expanded glacial sea ice cover (Fig. 5, C and D). This increase is opposite to the prediction of Ito and Follows (*30*), who ignored the upwelling of regenerated carbon through the base of the mixed layer. Additional sources of regenerated carbon may come from biological production, export, and remineralization. Together, all these processes contribute to maintaining a finite disequilibrium fraction even at large τ_air-sea_/τ_gas_.

**Fig. 5.**
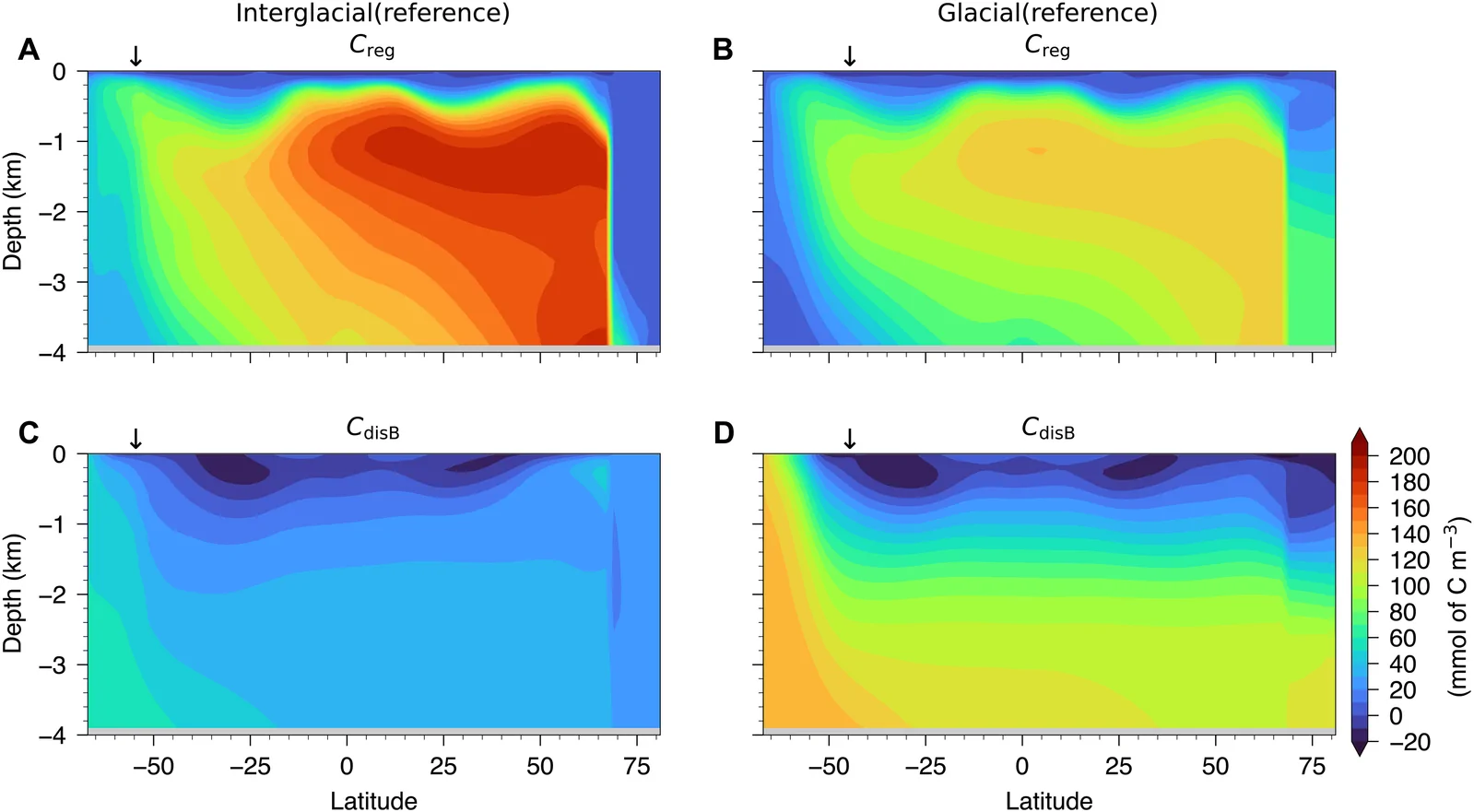
Interglacial and glacial regenerated and biological disequilibrium carbon reservoirs. Final states of Interglacial(reference) (left column) and Glacial(reference) (right column) for the global zonally integrated (**A** and **B**) regenerated, *C*_reg_, and (**C** and **D**) biological disequilibrium, *C*_disB_, reservoirs. Contour intervals are 10 mmol of C m^−3^. Downward black arrows indicate the maximum zonally averaged Antarctic sea ice edge.

#### The role of deep-ocean ventilation

Figure 4 provides compelling evidence that shorter air-sea equilibration times relative to the gas equilibration timescale, driven by the combined effects of sea ice expansion and circulation changes, limit the period during which surface waters outgas upwelled carbon and thereby systematically enhance the storage of biological disequilibrium carbon. As a result, lower τ_air-sea_/τ_gas_ ratios imply increasingly isolated deep waters and should therefore be closely linked to both *C*_disB_ and deep-ocean ventilation.

To quantify deep-ocean ventilation, we use three passive age tracers following Nadeau *et al.* (*40*), which age by 1 year every year in the ocean interior but are restored to zero at the surface in ice-free regions using idealized spatially uniform piston velocities representative of relatively fast (O_2_-like; 10 m day^−1^), intermediate (CO_2_-like; 1 m day^−1^), and slow (δ^13^C-like; 0.1 m day^−1^) gas equilibration timescales. The tracers obey no-flux conditions under full sea ice concentration, so waters upwelling beneath sea ice continue to age. As sea ice concentration decreases, the piston velocity is scaled by the open-water fraction to account for partial air-sea exchange.

Our results reveal a positive correlation between *C*_disB_ and ventilation age (Fig. 6), independent of the piston velocity, demonstrating that biological disequilibrium carbon storage increases as deep waters become more isolated from the atmosphere. The O_2_-like tracer exhibits slightly more scatter, likely because its rapid restoring reduces its sensitivity to small differences in surface exposure.

**Fig. 6.**
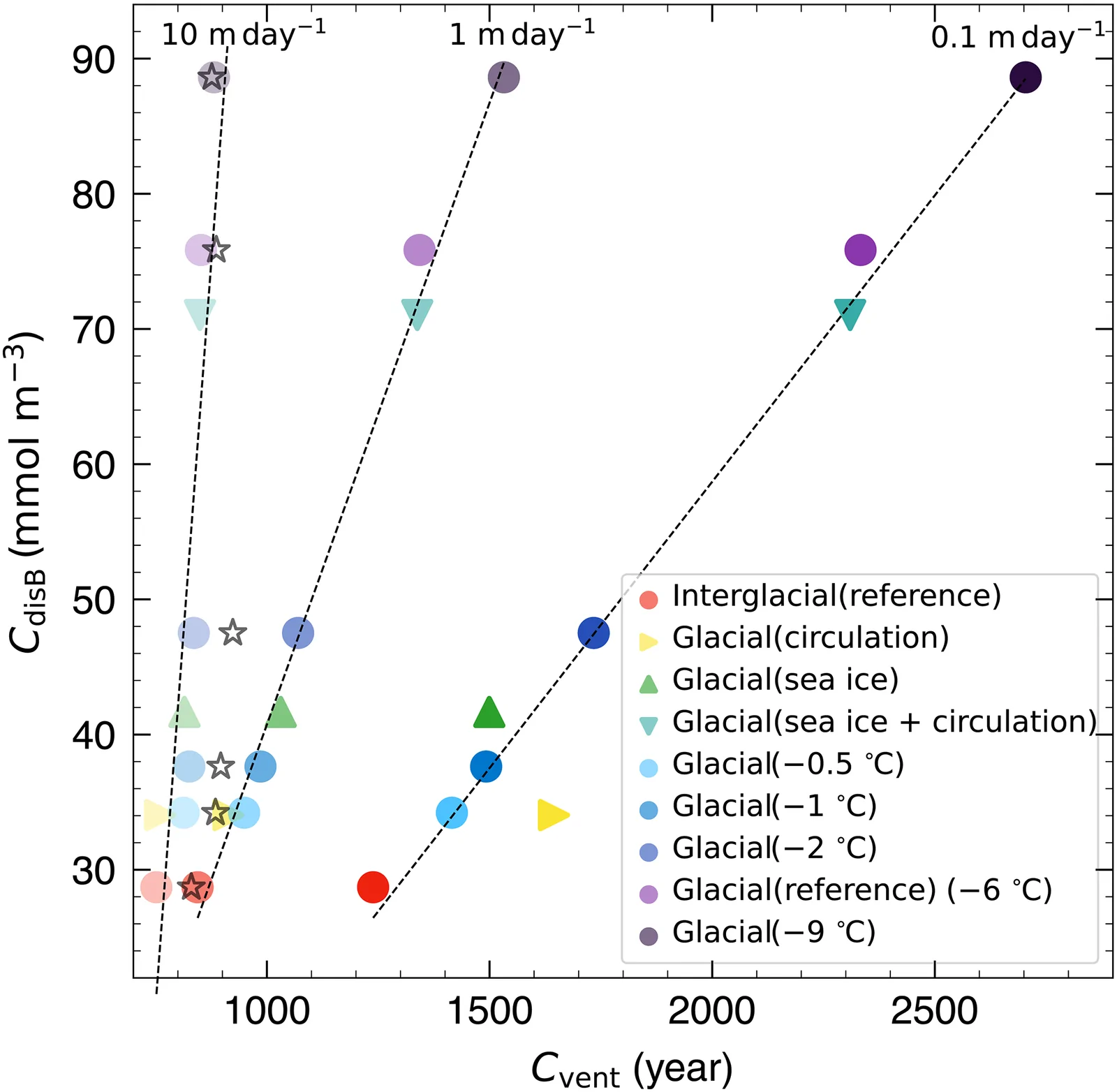
Dependence of biological disequilibrium carbon on ventilation age. Global mean biological disequilibrium carbon concentration, *C*_disB_, as a function of the global-average water mass ventilation age, *C*_vent_, computed with different restoring timescales. Dashed lines indicate linear fits labeled by restoring time. Stars denote the integrated ideal age, *C*_ideal_. Values of *C*_vent_ and *C*_ideal_ are given in table S3.

In contrast, an ideal age tracer that is reset to zero everywhere at the surface, including beneath sea ice, shows no consistent relationship with *C*_disB_ (Fig. 6). Because it is reset to zero even under full sea ice cover, it cannot record the degree of atmospheric isolation experienced by upwelling waters and therefore fails to capture differences in deep-water ventilation across the simulations. This is further supported by a sensitivity simulation in which sea ice is made permeable to light and gas exchange (figs. S8H and S9G): In this case, the Δ*C*_disB_ signal is largely erased and the associated Δ*P*co_2_^atm^ drawdown substantially reduced compared to the full glacial scenario. This underscores the importance of using a ventilated age tracer, as opposed to an ideal age tracer, to accurately quantify deep-water ventilation and capture the insulating effects of sea ice cover on air-sea exchange. Our simulations suggest that models lacking explicit preformed tracers may still estimate *C*_disB_ reliably using water-mass age, provided that the tracer accurately captures air-sea exchange limitations.

Large water-mass ages are often interpreted as reflecting enhanced regenerated carbon storage based on the classical view that poorly ventilated waters accumulate *C*_reg_ over time. Our results demonstrate the opposite: Older waters, as found in Glacial(reference), are instead associated with reduced regenerated carbon and a substantial increase in biological disequilibrium carbon (Fig. 3). Hence, in our simulations, large water-mass ages primarily reflect the degree of atmospheric isolation experienced by surface waters before subduction, rather than the accumulation of regenerated carbon in the ocean interior.

The substantial decrease in air-sea equilibration time, as well as the resulting isolation of deep waters, drives the sharp increase in *C*_disB_ in Glacial(sea ice + circulation) (Fig. 3B). This increase, together with a circulation-driven carbon gain in $\Delta C_{\text{sol}}\left(A_{\text{pre}}\right)$, fully offsets the substantial carbon loss in Δ*C*_reg_ also linked to circulation changes (Fig. 3, B and E, and fig. S6, D and F). As a result, the net carbon gain in Δ*C*_bgc_ reaches a magnitude comparable to that in Glacial(reference) (Fig. 2, A and B). Despite this amplified biogeochemical carbon gain, the net *P*co_2_^atm^ drawdown in Glacial(sea ice + circulation) accounts for less than half of that in Glacial(reference) (Fig. 2, A and B), due to a decrease in Δ*C*_disP_ caused by the expanded sea ice cover. As in Glacial(sea ice), the greater sea ice extent limits CO_2_ uptake by cold high-latitude waters, enhancing surface water undersaturation even as upwelling shifts equatorward under glacial circulation. We next isolate the effect of temperature to assess its individual contribution to the carbon budget.

### Ocean carbon storage in response to cooling: Amplified physical carbon storage due to solubility and physical disequilibrium

Cooling enhances the solubility of CO_2_ in seawater by shifting the thermodynamic balance of the carbon system chemistry toward bicarbonate and carbonate ions, thereby promoting uptake of CO_2_ from the atmosphere. Our Glacial(temperature) simulation produces the largest *P*co_2_^atm^ drawdown, accounting for 90% of that seen in Glacial(reference) (Fig. 2E). While CO_2_ uptake in Glacial(reference) is dominated by biogeochemical carbon storage in Δ*C*_bgc_, the carbon storage in Glacial(temperature) is almost entirely driven by physical processes, as reflected in a marked increase in Δ*C*_phys_ (Fig. 2, A and E). This contrast is further illustrated by the distinct carbon budget fingerprints of the two simulations (Fig. 3, A and C).

About half of the *P*co_2_^atm^ drawdown in Glacial(temperature) arises from the direct effect of cooling on solubility (Figs. 3C and 7B). The remainder is associated with better physical equilibration between the ocean and atmosphere (Figs. 3C and 7C). This effect is particularly pronounced in the high-latitude regions where deep waters form, both in the Southern Ocean and the North Atlantic. There, glacial temperatures exhibit only modest cooling relative to the interglacial state, whose waters were already near freezing (Fig. 7A), strongly reducing physical undersaturation and yielding large positive Δ*C*_disP_ anomalies (Fig. 7C). These results align with Khatiwala *et al.* (*16*), who showed that reduced undersaturation in cold high-latitude waters enhances physical disequilibrium carbon storage beyond what solubility changes alone would predict.

**Fig. 7.**
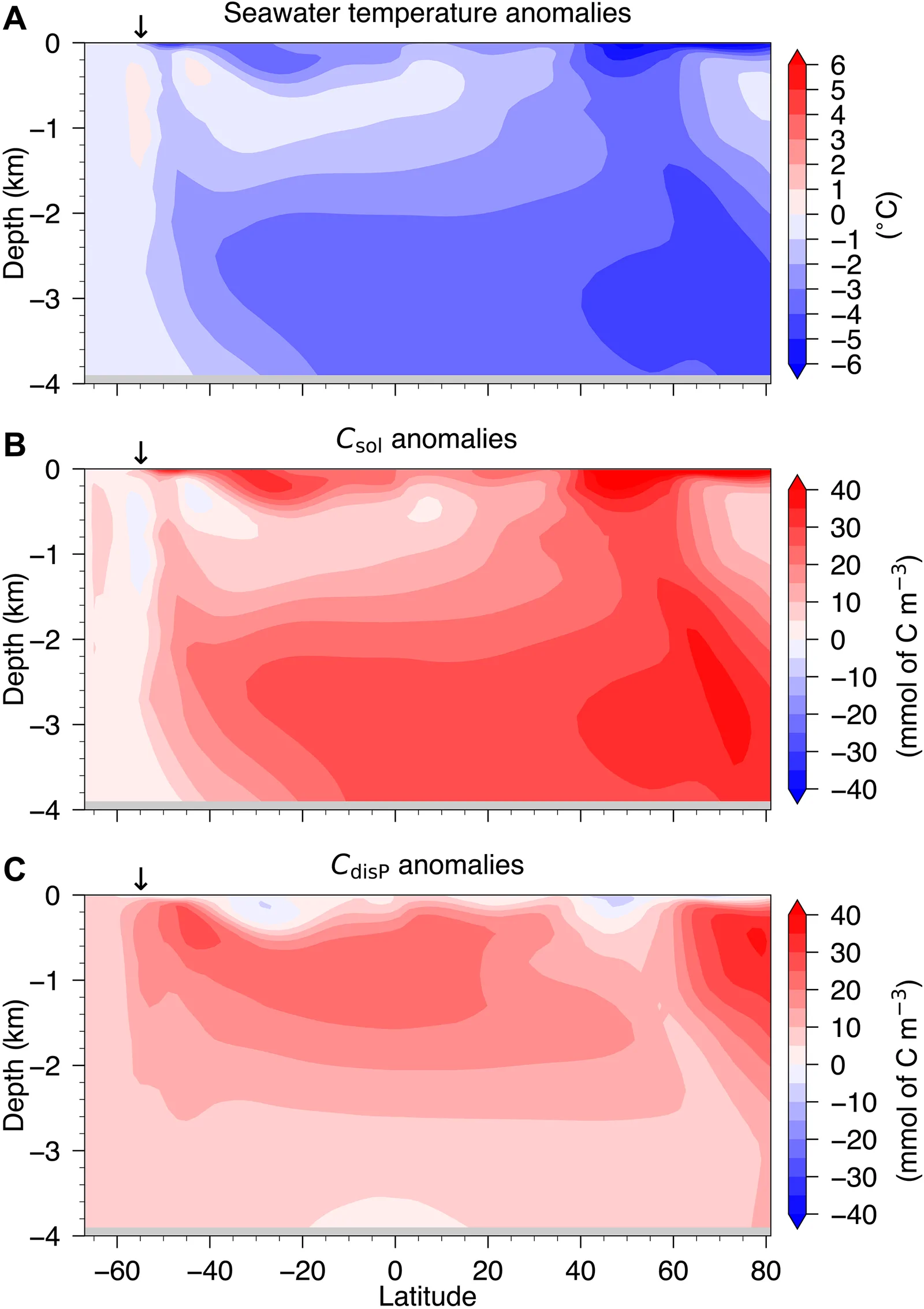
Effect of seawater cooling alone on carbon storage. Anomalies of Glacial(temperature) relative to Interglacial(reference) for (**A**) seawater temperature, (**B**) the solubility reservoir, Δ*C*_sol_, evaluated at the prognostic *P*co_2_^atm^ value of Interglacial(reference), and (**C**) the physical disequilibrium reservoir, Δ*C*_disP_, all zonally integrated over the Atlantic. Contour intervals are 1°C for temperature and 5 mmol of C m^−3^ for Δ*C*_sol_ and Δ*C*_disP_. Downward black arrows indicate the maximum zonally averaged interglacial Antarctic sea ice edge.

The combined effects of cooling with circulation and with sea ice were also examined (figs. S8, F and G, and S9, E and F). Although Glacial(sea ice + temperature) yields a *P*co_2_^atm^ drawdown comparable to Glacial(reference), the underlying processes leading to carbon storage in these two experiments again differ from Glacial(reference), as indicated by their distinct carbon fingerprints.

Together, these results emphasize the distinct processes by which cooling increases ocean carbon storage, in contrast to the combined effects of sea ice and circulation changes. While the biological carbon signal in Glacial(reference), dominated by *C*_disB_, arises from the combination of Antarctic sea ice expansion and circulation changes, the full glacial *P*co_2_^atm^ drawdown nevertheless requires the addition of cooling. By increasing carbon solubility and subsequently reducing physical disequilibrium (i.e., less negative anomalies), cooling acts as a necessary complement to the biogeochemical processes driving glacial carbon storage.

## DISCUSSION

In a series of idealized numerical simulations using an ocean–sea ice biogeochemical model, we investigated how key physical drivers of CO_2_ uptake in glacial climates, namely, extended Antarctic sea ice cover, glacial ocean circulation, and cooler seawater temperature, individually and in combination, alter ocean carbon storage. Only the combination of reconfigured ocean circulation and Antarctic sea ice expansion was able to capture the biologically sequestered carbon increase observed in the Glacial(reference) simulation. Circulation and sea ice, considered individually, led to minor CO_2_ outgassing, while cooling alone resulted in a physical rather than biological disequilibrium carbon gain (Figs. 2 and 3). These results highlight the critical role of their combined effects, rather than their isolated contributions, in shaping glacial carbon storage, which is linked to changes in air-sea equilibration time in the Southern Ocean.

The role of sea ice and circulation in glacial carbon storage has been highlighted in previous studies (*31*, *40*, *60*). Recently, Stein *et al.* (*31*) linked a 10-fold decrease in Southern Ocean air-sea equilibration time to a 40-μatm *P*co_2_^atm^ reduction across eight glacial cycles. Our results identify the underlying mechanism: Extensive Antarctic sea ice and glacial overturning circulation act together to reduce air-sea equilibration time, limiting atmosphere-ocean exchange and trapping upwelled biogenic carbon. Rather than being outgassed, this carbon is resubducted into the deep ocean, where enhanced isolation of abyssal waters leads to a sharp increase in biological disequilibrium carbon sequestration.

Crucially, while it is common to examine sea ice and ocean circulation in isolation to assess their impact on carbon storage (*16*, *19*, *57*, *65*, *66*), our results demonstrate that treating them separately misses a key aspect of the glacial signal. Their combination gives rise to nonlinear interactions that substantially amplify both *C*_disB_ and atmospheric CO_2_ drawdown, as shown by the discrepancy between their combined and individually summed effects (Fig. 8). This nonadditive behavior reinforces previous suggestions (*14*, *20*, *22*) that individual mechanisms should not be examined in isolation, as their effects depend on how they modulate one another.

**Fig. 8.**
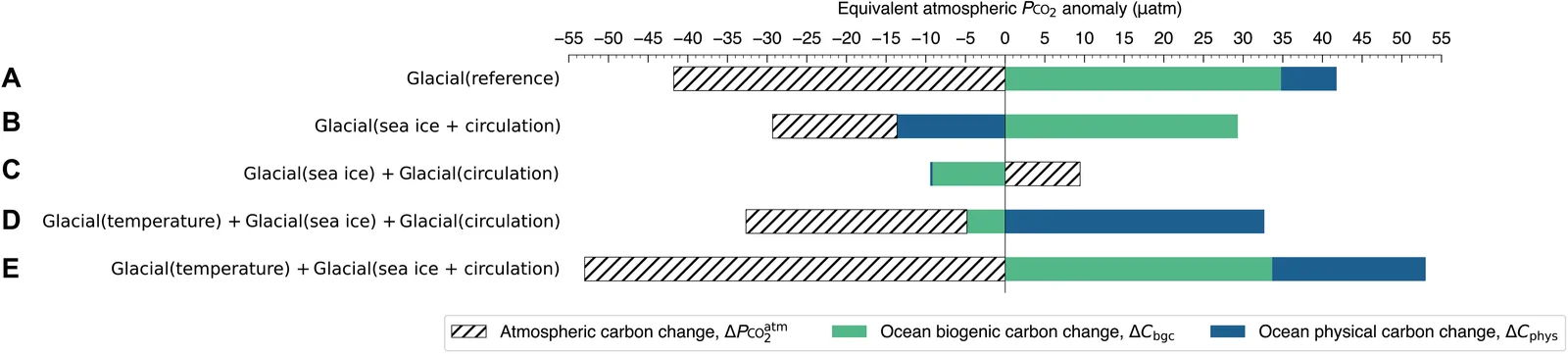
Linear superpositions of driver simulations for atmospheric and oceanic carbon anomalies. Same as Fig. 2, but for different linear superpositions of the driver experiments (**C** to **E**) to highlight nonlinear interactions. Anomalies from Glacial(reference) (**A**) and Glacial(sea ice + circulation) (**B**) are shown for comparison.

### The role of deep ocean mixing

This study highlights the importance of atmosphere-ocean exchange and biological disequilibrium changes in carbon sequestration in the glacial Southern Ocean. However, the ocean vertical mixing profile also critically modulates *P*co_2_^atm^ changes between glacial and interglacial climates (*29*, *40*, *60*), motivating a discussion of how it shapes the solutions we discussed so far.

No long-term carbon sequestration can be achieved if the mid-depth and abyssal overturning branches remain connected in a single loop, as in Interglacial(reference), where deep waters are returned through levels of strong vertical mixing—the prescribed vertical diffusivity decreases exponentially away from the seafloor (*67*) (fig. S1E). In such a circulation, all abyssal waters connect to the mid-depth cell, ultimately releasing their excess carbon as they reach the surface in the Southern Ocean and flow northward, away from sea ice around Antarctica.

In contrast, the Glacial(reference) simulation features a shoaled mid-depth cell that spans depths of weak vertical mixing and is effectively decoupled from the abyssal cell, consistent with proxy evidence (*55*, *59*). Abyssal waters remain confined below 2000 m, except where they outcrop in the Southern Ocean and flow southward beneath sea ice. This decoupling profoundly alters the carbon budget: Abyssal waters can no longer release the majority of their biological carbon to the atmosphere, as they outcrop under sea ice. Instead, they continue to accumulate carbon raining from the productive surface until a balance is achieved between the accumulation and the diffusion of DIC from the abyssal cell to shallower waters. Because diffusion of DIC is proportional to the product of vertical diffusivity and the vertical DIC gradient, if mixing is extremely weak at the top of the abyssal cell, as is the case in glacial climates, maintaining steady-state atmosphere-ocean carbon partitioning requires a strong DIC gradient between the abyssal cell and overlying waters.

Numerous studies have linked weak vertical mixing to deep-ocean carbon storage (*29*, *40*, *68*). We complement these prior studies by highlighting that an isolated abyssal cell shortens the Southern Ocean air-sea equilibration time, thereby reducing air-sea fluxes and enhancing biological disequilibrium carbon storage. The importance of mixing in trapping carbon in the deep ocean is clearly demonstrated by the Glacial(sea ice) simulation, which imposed expanded Antarctic sea ice coverage over the Southern Ocean while maintaining the interglacial circulation in which the abyssal and mid-depth cells are connected. In this configuration, carbon-rich abyssal waters join the mid-depth cell and flow northward, after outcropping at the surface, away from the Antarctic sea ice cover, leading to degassing and a net carbon loss from the ocean. Together, these results highlight the dual role of circulation and vertical mixing in modulating glacial carbon storage: By both limiting physical connectivity and weakening vertical diffusion, the glacial ocean effectively traps carbon-rich waters at depth.

### Link to glacial δ^13^C distributions

Numerous modeling and proxy-based studies have examined glacial carbon storage in the context of coupled changes in ocean circulation, sea ice extent, and water-mass properties (*69*–*72*). The individual effects of these processes cannot easily be disentangled in these studies, as they jointly change ocean ventilation, stratification, and the distribution of carbon isotopes. Many of them (*69*–*71*) conclude that a slowdown or a shoaling of the AMOC contributed substantially to the glacial CO_2_ drawdown by reducing deep-water renewal, consistent with the coevolution of negative deep-ocean δ^13^C and older Δ^14^C signals. Our experiments extend this view by explicitly isolating and recombining the effects of ocean circulation and Antarctic sea ice expansion, thereby highlighting how sea ice expansion amplifies the impact of circulation reorganization and jointly promotes reduced ventilation and enhanced carbon sequestration.

Moreover, our results align with the mechanism proposed by Muglia and Schmittner (*55*), who emphasized that apparent radiocarbon ages in the deep ocean largely reflect Southern Ocean surface reservoir ages, controlled by limited air-sea exchange, rather than slow interior transport. In our simulations, the biological disequilibrium carbon reservoir, *C*_disB_, and water-mass age increase, as the air-sea equilibration time, τ_air-sea_, decreases, consistent with this interpretation: Shorter exposure of surface waters to the atmosphere limits equilibration, allowing isotopic and chemical disequilibria to grow and enhancing carbon storage upon subduction.

Together, these findings point to the predominance of biological disequilibrium carbon in our simulations, in agreement with recent modeling and proxy evidence (*15*, *73*, *74*), which suggest that the negative deep-ocean δ^13^C signal observed in glacial records (*59*) primarily reflects enhanced biological disequilibrium carbon storage rather than increased regenerated carbon. Recent proxy-constrained estimates also suggest comparable contributions from disequilibrium and regenerated carbon storage at the LGM (*75*).

This implies that glacial carbon storage may have relied more on the accumulation of preformed nutrients and carbon linked to greater *C*_disB_, rather than on enhanced *C*_reg_, marking a shift away from the traditional emphasis on regenerated carbon reservoir and the biological pump. We propose a revised view of how glacial ocean carbon storage is attributed to the biological pump, with important implications for proxy interpretation. Although Vollmer *et al.* (*66*) and Omta *et al.* (*74*) arrive at similar estimates of total biologically stored carbon at the LGM, their mechanistic interpretation differs. Our results support the interpretation of Omta *et al.* (*74*), who attribute the signal to enhanced biological disequilibrium carbon storage, rather than the view of Vollmer *et al.* (*66*), who infer a stronger biological pump.

Vollmer *et al.* (*66*) inferred a stronger glacial biological pump based on AOU as a proxy for respired carbon. However, AOU assumes full oxygen equilibration at the surface, a condition unlikely to hold under glacial conditions, particularly in regions with extensive sea ice cover that restricts air-sea gas exchange while experiencing strong upwelling of undersaturated deep waters. To address the inconsistency between our finding of reduced *C*_reg_ and the increased AOU-based remineralization signal in glacial reconstructions [e.g., (*66*)], we assessed the effect of oxygen disequilibrium on AOU-based estimates of preformed nutrients.

In our simulations, the discrepancy between diagnosed preformed phosphate and AOU-inferred preformed phosphate increases proportionally with oxygen undersaturation, defined as ${O_{2}}^{\text{pre}}-{O_{2}}^{\text{sat}}$, demonstrating that reduced air-sea exchange combined with upwelling of undersaturated waters under glacial conditions introduces substantial bias in AOU-based diagnostics (fig. S10). Across all simulations with extensive sea ice, AOU underestimates preformed O_2_ and consequently overestimates oxygen consumption due to remineralization, leading to artificially elevated estimates of biological pump strength. These results highlight the limitations of O_2_-based proxies under glacial conditions, where sea ice impedes gas exchange, and underscore the importance of accounting for disequilibrium when interpreting glacial proxy records.

### A coherent feedback to glacial CO_2_ drawdown

Our study relies on idealized steady glacial boundary conditions that isolate the physical effects of temperature, sea ice cover, and ocean circulation on ocean carbon storage. This choice intentionally suppresses seasonal variability to focus on the equilibrium mean state of the ocean–sea ice carbon system and its response to glacial forcing. We do not account for additional biogeochemical processes associated with established ice sheets and lowered sea levels, such as iron fertilization and whole-ocean changes in nutrient and alkalinity concentrations (*14*, *46*–*50*). Consequently, our simulations do not capture the full 90-μatm glacial *P*co_2_^atm^ drawdown, consistent with previous studies that have employed a similar approach (*22*, *29*). Overall, despite our idealized steady framework, our results remain consistent with those obtained from more comprehensive climate models such as Khatiwala *et al.* (*16*), supporting the robustness of our key findings.

An important implication of our work is that the amplification of biological ocean carbon storage under combined sea ice and circulation changes offers insights into the stability of glacial-interglacial CO_2_ cycles. As noted by Stein *et al.* (*31*), if physical and biogeochemical processes acted independently, then the relatively consistent magnitude of atmospheric CO_2_ variability over the past 800,000 years is difficult to explain. Our results suggest that these processes are linked through a feedback loop: Atmospheric cooling enhances Antarctic sea ice cover and reorganizes the overturning circulation, which together reduce air-sea equilibration time and strengthen biological disequilibrium carbon storage. The resulting atmospheric CO_2_ drawdown further amplifies cooling, reinforcing sea ice expansion, circulation changes, and enhanced disequilibrium carbon storage. This positive feedback is, however, moderated by the gradual diffusion of DIC from the deep ocean, which acts as a governor on long-term CO_2_ variability. When the insolation forcing strengthens, the feedback reverses: Reduced sea ice cover and reinvigorated circulation release stored carbon back to the atmosphere, promoting interglacial warming.

We lastly place our results in the context of available Southern Ocean proxy constraints. Proxy data, such as nitrate and nitrogen isotopes, suggest that nutrients at the surface of the Southern Ocean were depleted during the LGM, while the efficiency of surface productivity increased (*13*, *76*). Our simulations show a similar pattern for northward-flowing surface waters in the circumpolar channel, whereas the opposite trend occurs in the southward-flowing surface waters that sink and drive the abyssal overturning cell. Proxy coverage remains limited south of the polar front, which separates the northward- and southward-flowing surface waters in the Southern Ocean. However, if a similar proxy pattern were found south of the polar front, we may need to reconsider how sea ice affected surface productivity in the Southern Ocean. In our simulations, we ignored the seasonal cycle in radiative forcing. Future work could explore whether introducing a seasonal cycle in the forcing would result in large seasonal variations of sea ice extent, with sea ice expanding in winter but nearly absent in summer, leaving surface waters ice-free capped by a fresh surface layer instead. In such a scenario, biological activity could continue in summer (unlike in the permanent sea ice scenario considered in this paper that restricts light penetration), resulting in depleted surface nutrients and increased production efficiency. However, the strong stratification caused by the fresh surface summer waters would still isolate the surface waters from the interior and lead to a similar disequilibrium pattern as described in this paper, which only requires interior waters to be poorly ventilated in the Southern Ocean, regardless of the specific mechanism.

## Supplementary Materials

### This PDF file includes:

General circulation model configuration Carbon budget anomalies Carbon partitioning from preformed tracers and AOU-based estimates Tables S1 to S3 Figs. S1 to S10 References
