# Supplementary material for: Ocean carbon sequestration during glacial climates due to combined Antarctic sea ice expansion and ocean circulation change
Source: Sci Adv. 2026 Oct 21;12(43):eady6777. doi: 10.1126/sciadv.ady6777 (PMC13685917; doi:10.1126/sciadv.ady6777)
Supplement: Supplementary file 1 [file sciadv.ady6777_sm.pdf]

Science Advances
AAAS

Supplementary Materials for

Ocean carbon sequestration during glacial climates due to combined Antarctic sea ice expansion and ocean circulation change

Sandy Grégorio *et al.*

Corresponding author: Sandy Grégorio, sandy_gregorio@uqar.ca

Sci. Adv. **12**, eady6777 (2026)
DOI: 10.1126/sciadv.ady6777

This PDF file includes:

General circulation model configuration
Carbon budget anomalies
Carbon partitioning from preformed tracers and AOU-based estimates
Tables S1 to S3
Figs. S1 to S10
References

General Circulation Model configuration

The idealized two-basin geometry (Fig. S1A) spans $256°$ in longitude and extends from $70°\mathrm{S}$ to $84°\mathrm{N}$ in latitude. The narrower Atlantic-like basin is $64°$ wide, while the wider Indo-Pacific-like basin is $186°$ wide. A landmass between $70°\mathrm{N}$ and $84°\mathrm{N}$ mimics the reduced latitudinal extent of the North Pacific. The two basins are separated by two $2°$-wide land strips extending from the northern boundary to $50°\mathrm{S}$ and $38°\mathrm{S}$, representing Cape Horn and Cape Agulhas, respectively. The ocean bottom is flat and $4\ \mathrm{km}$ deep throughout the domain, except for a $2\ \mathrm{km}$-high sill in the circumpolar channel, simulating the Scotia Ridge at the latitudes of Drake Passage. Despite a smaller Southern Ocean, the total basin areas approximate those of the Atlantic and Indo-Pacific Oceans.

The model employs the non-linear equation of state from Jackett et al. (*84*) and assumes hydrostatic pressure. Momentum is dissipated via Laplacian viscosity with horizontal and vertical coefficients $\nu_\mathrm{H} = 2 \times 10^4\ \mathrm{m}^2\,\mathrm{s}^{-1}$ and $\nu_\mathrm{V} = 2 \times 10^{-3}\ \mathrm{m}^2\,\mathrm{s}^{-1}$, respectively. Baroclinic instability is parameterized following Gent and McWilliams (*37*) using a constant eddy diffusivity of $\kappa_\mathrm{GM} = 1000\ \mathrm{m}^2\,\mathrm{s}^{-1}$. Ocean convection is parameterized through convective adjustment, with an enhanced vertical diffusivity $\kappa_\mathrm{conv} = 10\ \mathrm{m}^2\,\mathrm{s}^{-1}$ prescribed where stratification is statically unstable. No-slip boundary conditions are applied at sidewalls, with a rigid-lid condition at the surface. A linear drag, with a coefficient of $2 \times 10^{-5}\ \mathrm{s}^{-1}$, is imposed on the $200\ \mathrm{m}$ deep bottom layer.

Momentum and sensible heat fluxes between the atmosphere and ocean are calculated using bulk formulas from Large and Yeager (*85*). The net atmospheric heat flux is given by the difference between the sensible heat flux and a parameterized radiative flux, given by $F_\mathrm{rad} = \sigma(\mathrm{SST}^4 - T_\mathrm{a}^4)$, where SST is the sea surface temperature, T_a is the prescribed atmospheric temperature (Fig. S1B), and $\sigma = 5.67 \times 10^{-8}\ \mathrm{W\,m^{-2}\,K^{-4}}$ is the Stefan-Boltzmann constant.

To simplify the boundary conditions, latent heat flux and solar radiation are ignored, following Jansen (*42*). To maintain a closed salt budget, the net precipitation per unit area is reduced at high latitudes in the Atlantic basin, compensating for its greater latitudinal extent. This adjustment ensures that, for a given longitude, total net precipitation remains consistent between the Atlantic and Pacific.

The biogeochemical module includes cycles of dissolved inorganic carbon (DIC), alkalinity (A_T), organic and inorganic phosphorus (DOP and $\mathrm{PO_4}$), and oxygen ($\mathrm{O_2}$). Preformed DIC (C_pre), alkalinity (A_pre) and phosphorus (${\mathrm{PO_4}}^\mathrm{pre}$) are explicitly simulated. The concentrations of the preformed tracers at the surface are set equal to those of the corresponding active tracers, after which they are passively advected and mixed in the ocean interior, once subducted.

Biological transformations are based on the Redfield stoichiometric ratios in the proportions $[C : N : P : O] = 117 : 16 : 1 : -170$ (*86*), with a prescribed inorganic to organic rain ratio of 7%. The air–sea $\mathrm{CO_2}$ flux is parameterized as the product of the gas transfer velocity, proportional to the square of the local wind speed (derived from the wind stress shown in Fig. S1D), the $\mathrm{CO_2}$ solubility coefficient, which depends on temperature and salinity, and the difference between atmospheric and surface ocean $p\mathrm{CO_2}$ (*71, 87*). In sea ice covered regions, this flux is scaled by the fraction of open water. More details on the biogeochemistry configuration are available in Lauderdale et al. (*26, 28*).

The model is first integrated using the interglacial atmospheric temperature profile in Fig. S1B. During the initial spin-up (16,000 years), $p{\mathrm{CO_2}}^\mathrm{atm}$ is restored to a prescribed value of 278 $\mu\mathrm{atm}$, while the carbon inventory in the model is allowed to adjust. A pair of simulations is then restarted for another 10,000 years, both with a well-mixed atmospheric carbon reservoir, but with biological productivity switched off for one of the simulations to isolate the physical contribution of the disequilibrium reservoir, C_disP. The biological contribution to disequilibrium carbon storage, C_disB, is found as the residual between the two simulations. These simulations

provide the initial conditions for the eight pairs of simulations listed in Table 1, each of which is run for another 10,000 years, as described in the Modeling approach subsection of the main text.

An additional pair of sensitivity experiments with sea ice permeable to light and air–sea fluxes is also conducted. The *glacial(permeable sea ice)* simulation is run as in *glacial(reference)*, including the 1-psu salinity increase, but initialized from *interglacial(permeable sea ice)*.

Carbon budget anomalies

Following Ito and Follows (*24*) and Goodwin et al. (*88*), the global inventory of carbon, $\sum C$, is given by:

$$\sum C = M \times p\mathrm{CO_2}^{\mathrm{atm}} + V \times (\underbrace{C_{\mathrm{sol}} + C_{\mathrm{dis}} + C_{\mathrm{reg}}}_{\mathrm{DIC}}), \tag{2}$$

where $M = 1.77 \times 10^{20}$ mol is the molar volume of the atmosphere and $V \sim 1.33 \times 10^{18}$ m^3 is the ocean volume. The terms C_{sol}, C_{dis}, and C_{reg} represent the globally averaged concentrations of carbon in the solubility, disequilibrium and regenerated reservoirs, respectively. Their sum corresponds to the globally averaged concentrations of DIC. As discussed in Ödalen et al. (*89*), a small residual difference between DIC and $C_{\mathrm{sol}} + C_{\mathrm{dis}} + C_{\mathrm{reg}}$ arises from organic matter and minor approximations in the theoretical formulations of C_{sol} and C_{reg}, leading to a negligible error ($< 0.01\%$).

Changes in the carbon reservoirs in response to glacial perturbations, compared to the interglacial state, are expressed as:

$$-\Delta p\mathrm{CO_2}^{\mathrm{atm}} = \frac{V}{M}\,(\Delta C_{\mathrm{sol}} + \Delta C_{\mathrm{dis}} + \Delta C_{\mathrm{reg}}), \tag{3}$$

which follows directly from Eq. 2 under total carbon conservation.

To better understand the processes driving changes in C_{sol}, we decompose ΔC_{sol} into con-

tributions from temperature, salinity, preformed alkalinity, and $p\mathrm{CO_2}^{\mathrm{atm}}$:

$$\Delta C_{\mathrm{sol}} \approx \Delta C_{\mathrm{sol}}(\theta) + \Delta C_{\mathrm{sol}}(S) + \Delta C_{\mathrm{sol}}(A_{\mathrm{pre}}) + \Delta C_{\mathrm{sol}}(p\mathrm{CO_2}^{\mathrm{atm}}). \tag{4}$$

Each contribution is evaluated following the one-at-a-time approach described in the Carbon framework subsection.

Building on the framework of Marzocchi and Jansen (*29*), and separating ΔC_{dis} into its physical and biological components, the global $\Delta p\mathrm{CO_2}^{\mathrm{atm}}$ is given by:

$$-\Delta p\mathrm{CO_2}^{\mathrm{atm}} = \alpha\,(\Delta C_{\mathrm{sol}}(\theta) + \Delta C_{\mathrm{sol}}(S) + \Delta C_{\mathrm{disP}} + \Delta C_{\mathrm{sol}}(A_{\mathrm{pre}}) + \Delta C_{\mathrm{disB}} + \Delta C_{\mathrm{reg}}), \tag{5}$$

where

$$\alpha = \frac{1}{\dfrac{M}{V} + \left.\dfrac{\partial C_{\mathrm{sol}}}{\partial p\mathrm{CO_2}^{\mathrm{atm}}}\right|_{\theta,\ S,\ A_{\mathrm{pre}}}}\ . \tag{6}$$

α is a scaling factor that accounts for the relative buffering effect of the ocean–atmosphere carbon exchange. In practice, α is diagnosed from the model results as:

$$\alpha = \frac{-\Delta p\mathrm{CO_2}^{\mathrm{atm}}}{\Delta C_{\mathrm{sol}}(\theta) + \Delta C_{\mathrm{sol}}(S) + \Delta C_{\mathrm{disP}} + \Delta C_{\mathrm{bgc}}}\ , \tag{7}$$

rather than computed analytically from Eq. 6.

Finally, the contributions of each carbon reservoir to $\Delta p\mathrm{CO_2}^{\mathrm{atm}}$ are given by:

$$(\Delta C_{\mathrm{sol}}(\theta))_{\mathrm{pCO_2}} = \alpha\,\Delta C_{\mathrm{sol}}(\theta), \tag{8a}$$

$$(\Delta C_{\mathrm{sol}}(S))_{\mathrm{pCO_2}} = \alpha\,\Delta C_{\mathrm{sol}}(S), \tag{8b}$$

$$(\Delta C_{\mathrm{disP}})_{\mathrm{pCO_2}} = \alpha\,\Delta C_{\mathrm{disP}}, \tag{8c}$$

$$(\Delta C_{\mathrm{sol}}(A_{\mathrm{pre}}))_{\mathrm{pCO_2}} = \alpha\,\Delta C_{\mathrm{sol}}(A_{\mathrm{pre}}), \tag{8d}$$

$$(\Delta C_{\mathrm{disB}})_{\mathrm{pCO_2}} = \alpha\,\Delta C_{\mathrm{disB}}, \tag{8e}$$

$$(\Delta C_{\mathrm{reg}})_{\mathrm{pCO_2}} = \alpha\,\Delta C_{\mathrm{reg}}. \tag{8f}$$

In these equations, the subscript $\mathrm{pCO_2}$ indicates that the contributions are expressed in units of $p\mathrm{CO_2}^{\mathrm{atm}}$, that is, as their equivalent impact on atmospheric $\mathrm{CO_2}$ rather than as carbon concentrations. In the main text, this subscript is omitted for clarity.

Carbon partitioning from preformed tracers and AOU-based estimates

We evaluate the simulated carbon reservoirs using both preformed tracers and Apparent Oxygen Utilization (AOU), to enable comparison with observational AOU-based estimates (*25*) (Table S1).

In the preformed tracer approach, C_{sol} is computed from simulated preformed alkalinity, A_{pre}. The regenerated component, C_{reg}, is diagnosed following Williams and Follows (*25*):

$$C_{\mathrm{reg}} = R_{\mathrm{CP}}\,(\mathrm{PO_4} - \mathrm{PO_4}^{\mathrm{pre}}) + \frac{1}{2}(A_{\mathrm{T}} - A_{\mathrm{pre}} + R_{\mathrm{NP}}\,(\mathrm{PO_4} - \mathrm{PO_4}^{\mathrm{pre}})), \tag{9}$$

where $R_{\mathrm{CP}} = 117$ and $R_{\mathrm{NP}} = 16$. The disequilibrium component, C_{dis}, is obtained as $C_{\mathrm{dis}} = C_{\mathrm{pre}} - C_{\mathrm{sol}}$.

In the AOU-based approach, the solubility component is recalculated using preformed alkalinity estimated via multiple linear regression, following Lauderdale et al. (*26*), using sea surface salinity and the conservative tracer PO at the surface, where $\mathrm{PO} = \mathrm{O_2} - R_{\mathrm{OP}}\,\mathrm{PO_4}$ represents the oxygen concentration corrected for the effects of respiration and remineralization of biogenic phosphate (*90*), with $R_{\mathrm{OP}} = -170$. The regression takes the form: $A_{\mathrm{pre,MRL}} = a + b\,S + c\,\mathrm{PO}$. The AOU-based estimate of the regenerated component (*25, 54*) is given by:

$$C_{\mathrm{reg,AOU}} = -R_{\mathrm{CO}}\,(\mathrm{O_2}^{\mathrm{sat}} - \mathrm{O_2}) + \frac{1}{2}(A_{\mathrm{T}} - A_{\mathrm{pre,MRL}} - R_{\mathrm{NO}}(\mathrm{O_2}^{\mathrm{sat}} - \mathrm{O_2})), \tag{10}$$

where $R_{\mathrm{CO}} = -106/170$ and $R_{\mathrm{NO}} = -16/170$. The Redfield ratios used in Eq. 10 are consistent with those employed in the data-based estimates from Williams and Follows (*25*). The disequilibrium reservoir is computed as $C_{\mathrm{dis,AOU}} = (\mathrm{DIC} - C_{\mathrm{reg,AOU}}) - C_{\mathrm{sol}}(A_{\mathrm{pre,MRL}})$.

The simulated carbon reservoirs estimated using AOU broadly agree with observations (Table S1). However, small differences between the preformed tracer and AOU estimates are highly significant. The AOU approach infers respiration by assuming that surface oxygen concentrations, and thus preformed oxygen, are close to saturation with the overlying atmosphere. This leads to an overestimation of respired carbon in high-latitude regions where sea ice and vigorous circulation stifle gas exchange. As a result, C_{reg} is overestimated, which in turn leads to an underestimation of C_{dis} (*16, 54*) (Table S1). In modeling studies, it is therefore more accurate to quantify carbon reservoirs directly from explicitly simulated preformed tracers (*16, 29, 91*).

Table S1: **Carbon partitioning in the interglacial simulation and observational estimates.** Carbon partitioning (PgC) for the interglacial(reference) state, with observational estimates taken from Table 13.1 of Williams and Follows (*25*). While Williams and Follows (*25*) refer to the saturated component, C_{sat}, we use the term solubility component, C_{sol}, which is conceptually equivalent. Model-derived values based on preformed tracers are computed using the model's stoichiometric ratio $[C : N : P : O] = 117 : 16 : 1 : -170$, whereas AOU-based estimates, both from the model and from observations, follow the canonical Redfield ratio $[C : N : P : O] = 106 : 16 : 1 : -170$. Note that the data-based estimate for C_{dis} includes a contribution from anthropogenic carbon, which is not taken into account in our model.

	interglacial(reference)		observations
DIC	35785		35538
	Preformed tracers	AOU estimate	AOU estimate
C_{sol}	33736	33694	33330
C_{reg}	1950	2068	2174
C_{dis}	102	23	38
C_{disP}	-356	-378	-
C_{disB}	$+458$	$+401$	-

Table S2: **Atmospheric and oceanic carbon pool anomalies in response to glacial perturbations.** Atmospheric and oceanic carbon pool anomalies (μatm) in response to the physical glacial perturbations relative to *interglacial(reference)* or *interglacial(permeable sea ice)*.

**Glacial perturbations**	$\Delta p CO_2^{atm}$	$\Delta C_{sol}(\theta)$	$\Delta C_{sol}(S)$	ΔC_{disP}	$\Delta C_{sol}(A_{pre})$	ΔC_{disB}	ΔC_{reg}
glacial(−0.5 °C)	−8.0	+7.5	−5.5	−1.8	−1.1	+7.4	+1.5
glacial(−1 °C)	−11.6	+9.9	−5.4	−3.3	+0.4	+11.9	−1.9
glacial(−2 °C)	−17.4	+9.2	−5.2	−0.2	+7.0	+24.4	−17.8
glacial(reference)(−6 °C)	−41.7	+19.0	−4.7	−7.3	+19.1	+58.6	−43.0
glacial(−9 °C)	−50.0	+21.8	−4.4	−10.2	+25.1	+73.3	−55.6
glacial(permeable sea ice)	−9.0	+19.0	−4.8	+10.7	+20.9	+7.7	−44.5
glacial(sea ice+temperature)	−40.6	+19.6	0.0	+5.9	−0.1	+20.5	−5.3
glacial(sea ice+circulation)	−15.7	0.0	0.0	−13.6	+19.4	+53.2	−43.3
glacial(circulation+temperature)	−14.1	+19.6	0.0	+9.9	+21.6	+8.7	−45.7
glacial(temperature)	−37.3	+19.8	0.0	+13.1	0.2	+4.4	−0.2
glacial(circulation)	+7.1	0.0	0.0	+13.7	+25.9	+8.2	−54.9
glacial(sea ice)	+2.4	0.0	0.0	−14.0	−0.1	+17.1	−5.4

Table S3: **Air–sea equilibration time and water mass ages.** Air–sea equilibration time in the Southern Ocean, $\tau_{air-sea}$, CO_2 equilibration timescale in the Southern Ocean, τ_{gas}, global-average water mass ventilation age with $\delta^{13}C$-, CO_2-, and O_2-like restoring timescales, $C_{vent,\delta^{13}C}$, C_{vent,CO_2}, and C_{vent,O_2}, together with the ideal age, C_{ideal}. All values are in years.

**Simulations**	$\tau_{air-sea}$	τ_{gas}	$C_{vent,\delta^{13}C}$	C_{vent,CO_2}	C_{vent,O_2}	C_{ideal}
interglacial(reference)	5.51	4.00	1239	844	752	830
glacial(−0.5 °C)	2.54	4.29	1415	949	813	885
glacial(−1 °C)	1.69	3.90	1492	986	826	896
glacial(−2 °C)	0.79	4.32	1734	1070	836	923
glacial(reference)	0.24	3.55	2333	1343	852	886
glacial(−9 °C)	0.17	3.37	2704	1532	881	876
glacial(circulation)	1.76	3.55	1645	913	760	–
glacial(sea ice)	0.76	4.00	1499	1031	815	–
glacial(sea ice+circulation)	0.24	3.55	2309	1338	850	–

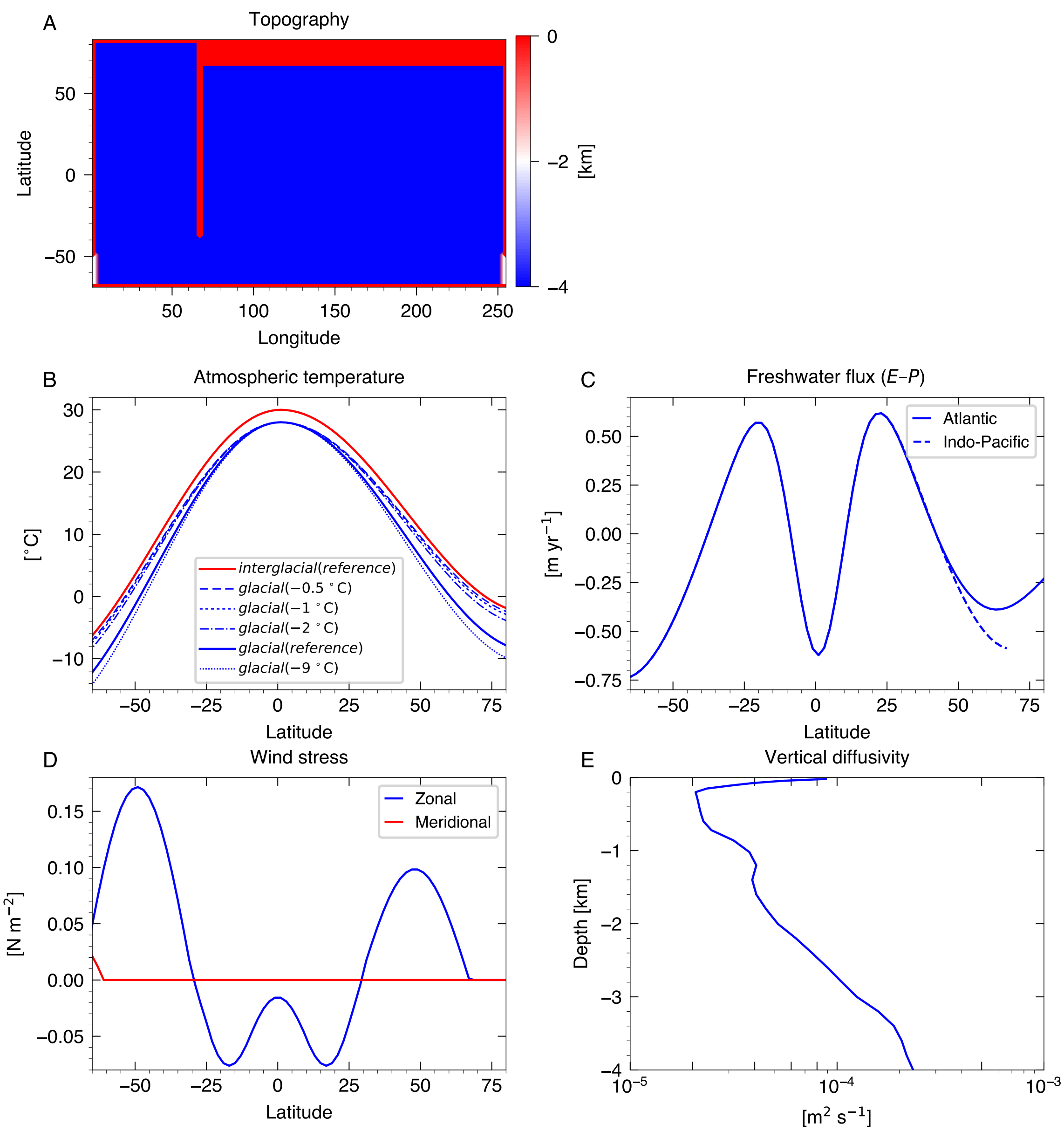

Fig. S1: **Model configuration and atmospheric forcing.** (A) Model topography. (B) Atmospheric temperature profiles for *interglacial(reference)* (red solid line), *glacial(*$-0.5\ °C$*)* (blue long-dashed line), *glacial(*$-1\ °C$*)* (blue medium-dashed line), *glacial(*$-2\ °C$*)* (blue dash-dotted line), *glacial(reference)* (blue solid line) and *glacial(*$-9\ °C$*)* (blue dotted line). (C) Freshwater flux, defined as evaporation minus precipitation, in the Atlantic (solid line) and Indo-Pacific (dashed line). (D) Zonal (blue) and meridional (red) wind stresses. (E) Vertical profile of diapycnal diffusivity. All profiles are time-invariant and zonally uniform within each basin.

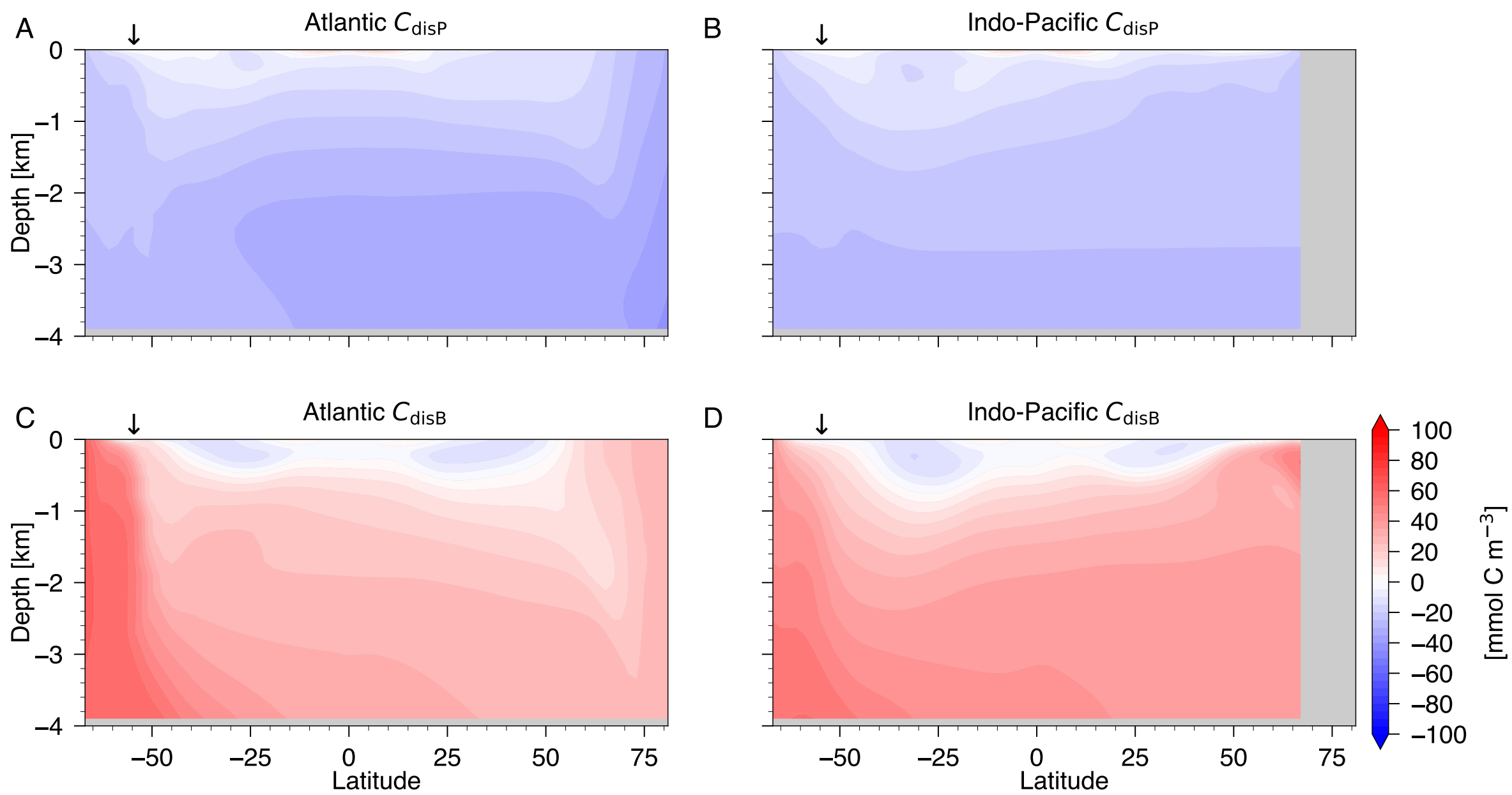

Fig. S2: **Interglacial physical and biological disequilibrium carbon reservoirs.** Final state of *interglacial(reference)* for the zonally integrated (A-B) physical, C_{disP}, and (C-D) biological, C_{disB}, disequilibrium reservoirs over the Atlantic (left column) and Indo-Pacific (right column). Contour intervals are 5 $\mathrm{mmol\,C\,m^{-3}}$. Downward black arrows indicate the maximum zonally averaged interglacial Antarctic sea ice edge.

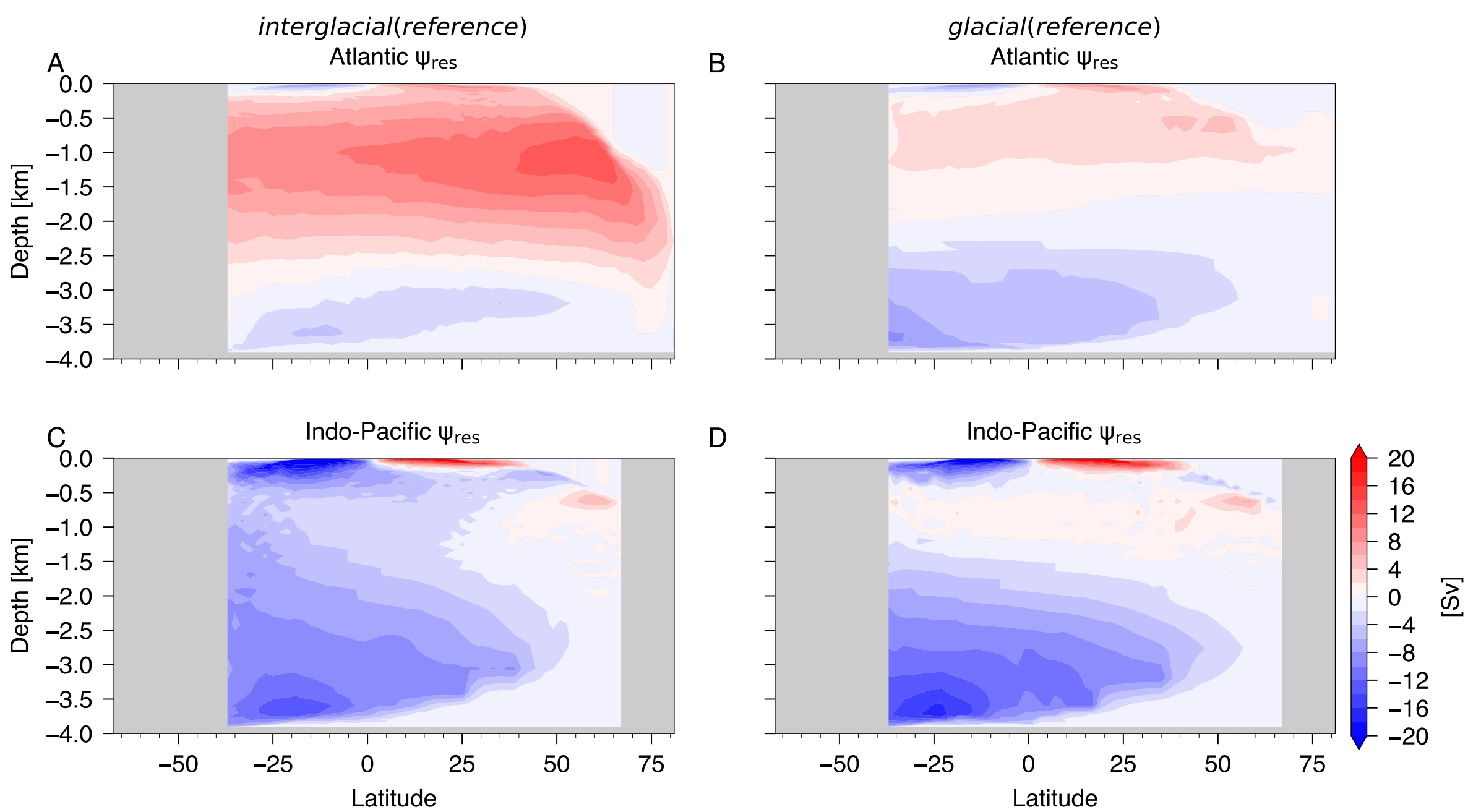

Fig. S3: **Residual overturning circulation under interglacial and glacial conditions.** Final states of *interglacial(reference)* (left column) and *glacial(reference)* (right column) for the zonally integrated residual meridional streamfunction, ψ_{res}, over the (A-B) Atlantic and (C-D) Indo-Pacific. ψ_{res} combines the Eulerian-mean and eddy-induced transports (*51*) and is computed in density space relative to 2000 dbar, then remapped to the zonally averaged depth of each density surface. Contour intervals are 2 Sv.

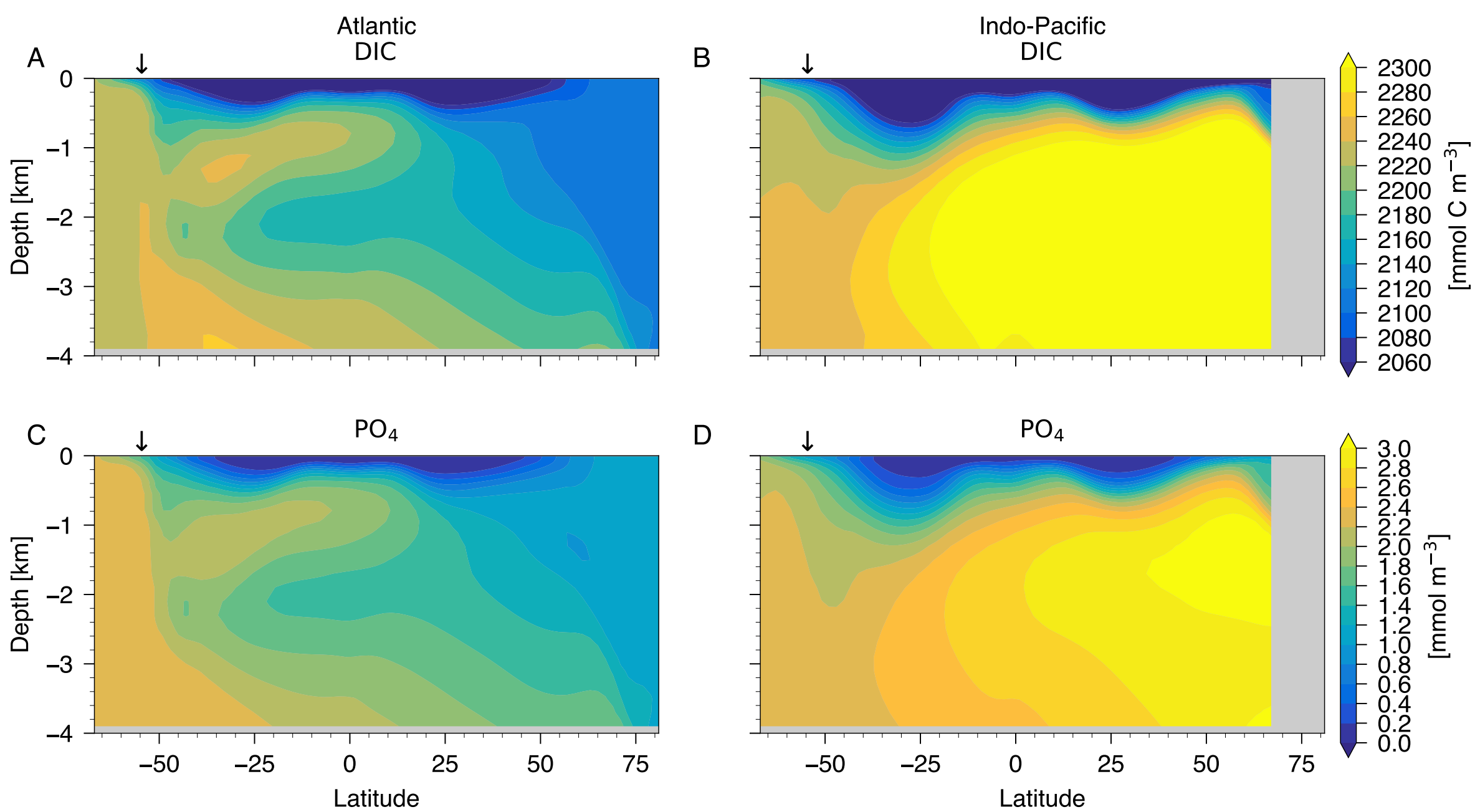

Fig. S4: **Interglacial distributions of dissolved inorganic carbon and phosphate.** Final state of *interglacial(reference)* for (A-B) DIC and (C-D) $\mathrm{PO_4}$, zonally integrated over the Atlantic (left column) and Indo-Pacific (right column). Contour intervals are 20 $\mathrm{mmol\,C\,m^{-3}}$ for DIC and 0.2 $\mathrm{mmol\,m^{-3}}$ for $\mathrm{PO_4}$. Downward black arrows indicate the maximum zonally averaged interglacial Antarctic sea ice edge.

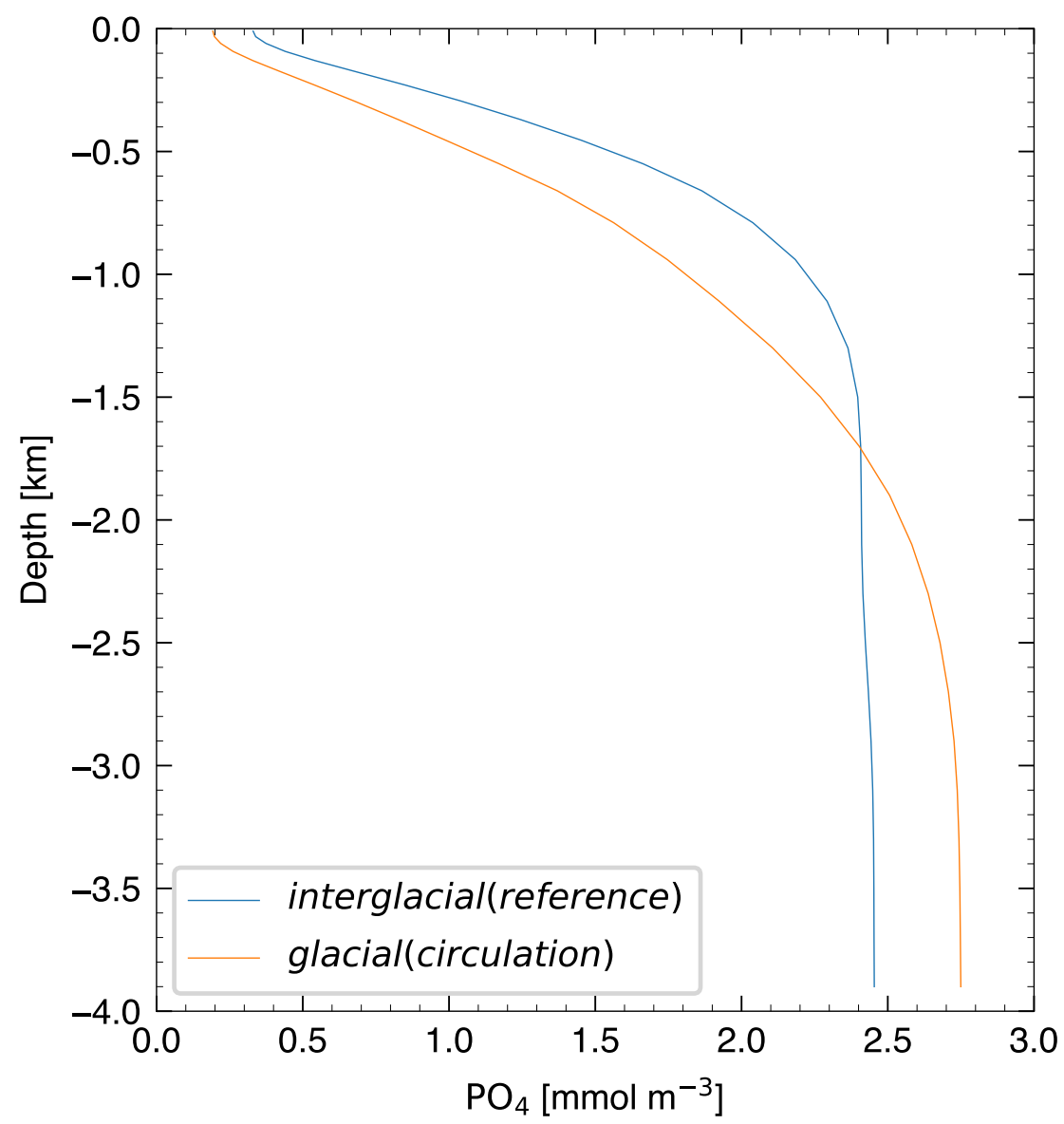

Fig. S5: **Global phosphate profiles for interglacial reference and glacial circulation-only simulations.** Global $\mathrm{PO_4}$ as a function of depth for *interglacial(reference)* and *glacial(circulation)*.

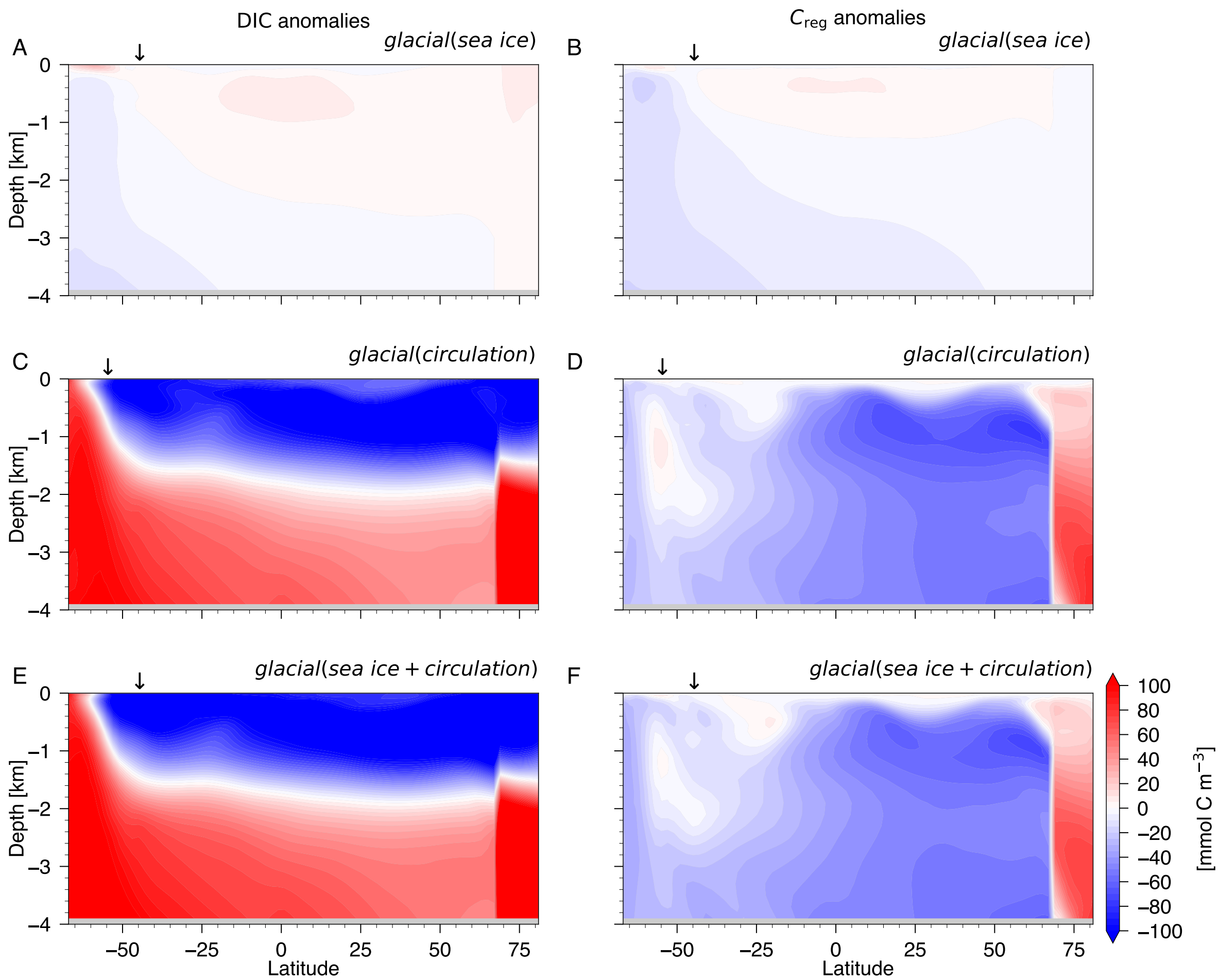

Fig. S6: **Anomalies of dissolved inorganic carbon and regenerated carbon in response to glacial perturbations.** Anomalies of dissolved inorganic carbon (left column) and the regenerated reservoir, C_{reg} (right column), zonally integrated over the full domain, for (A-B) *glacial(sea ice)*, (C-D) *glacial(circulation)* and (E-F) *glacial(sea ice+circulation)* relative to *interglacial(reference)*. Contour intervals are 5 $\mathrm{mmol\,C\,m^{-3}}$. Downward black arrows indicate the maximum zonally averaged interglacial (C, D) and glacial (A, B, E, F) Antarctic sea ice edge.

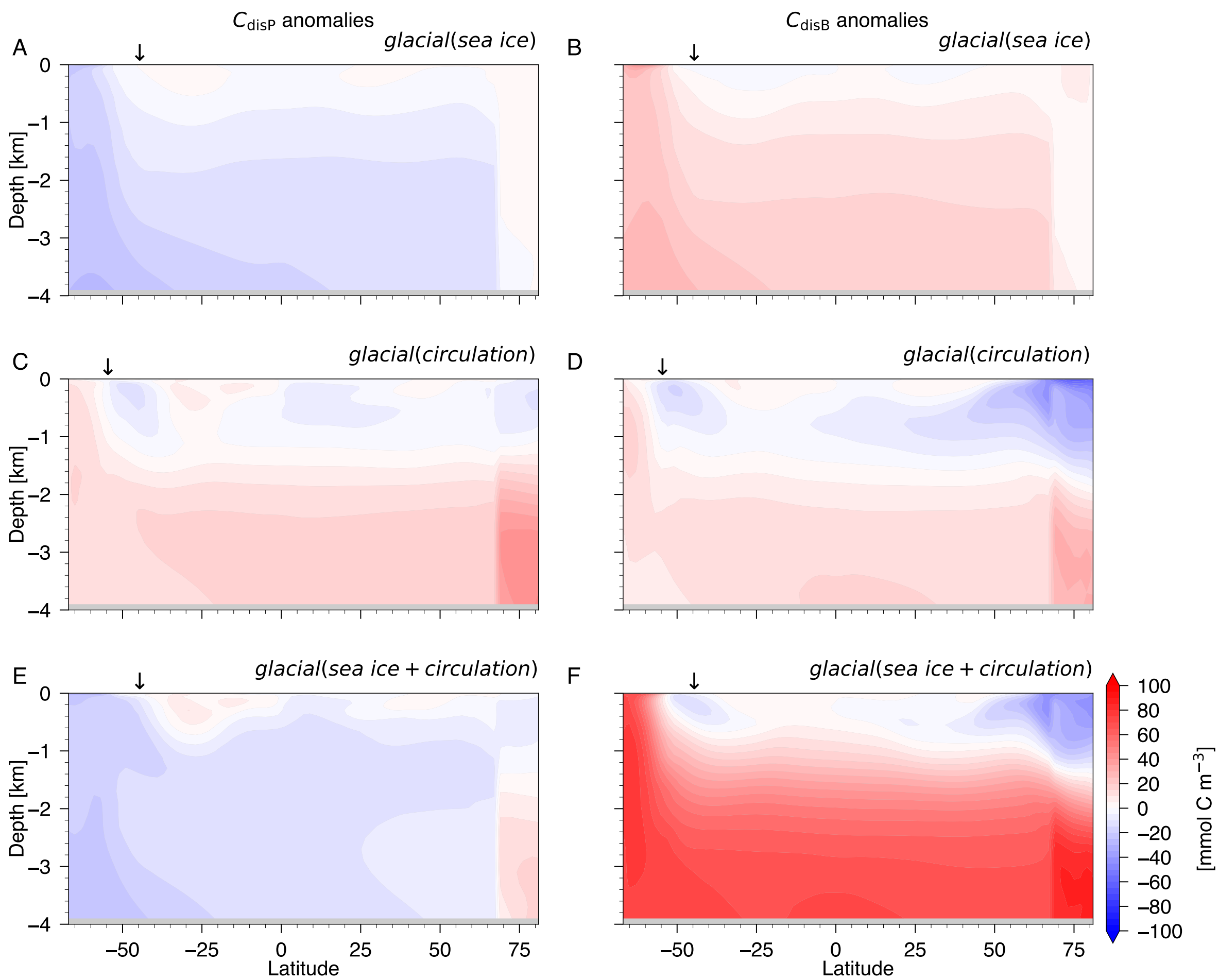

Fig. S7: **Anomalies of physical and biological disequilibrium carbon in response to glacial perturbations.** Same as Fig. S6, but for the physical, C_{disP} (left column), and biological, C_{disB} (right column), disequilibrium carbon reservoirs.

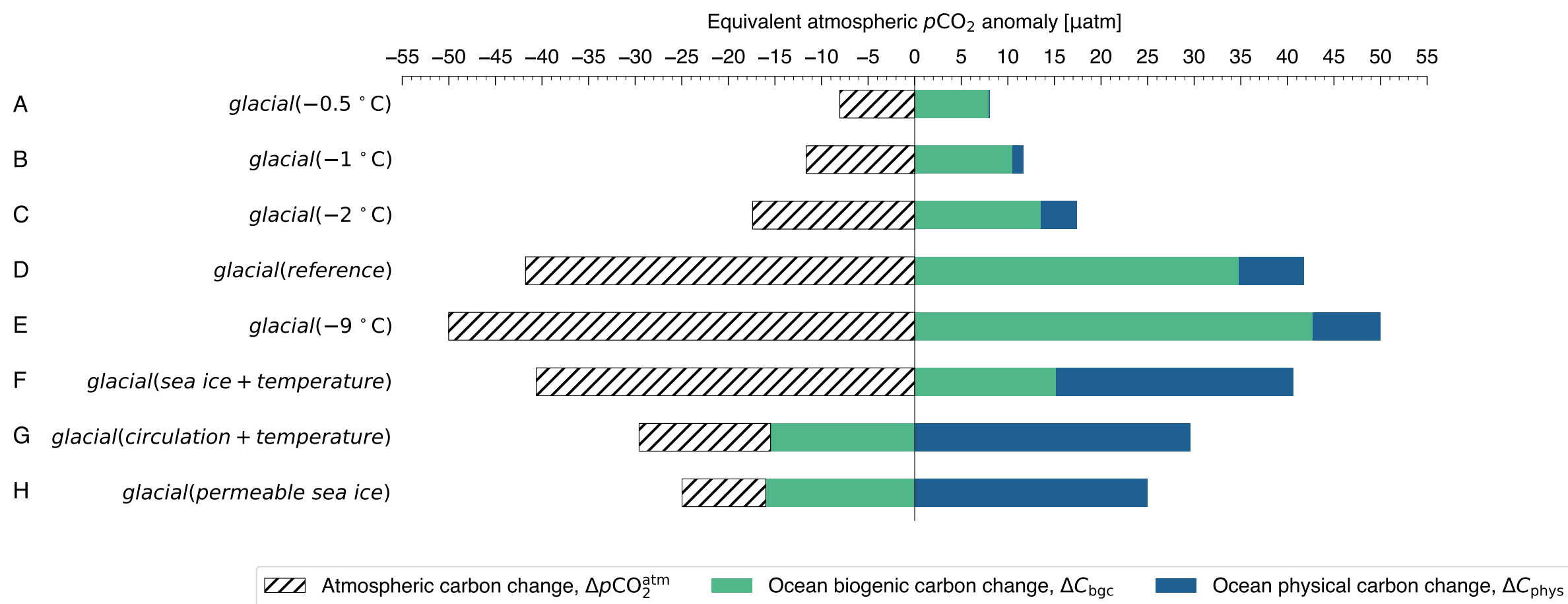

Fig. S8: **Partitioning of atmospheric and oceanic carbon anomalies in response to glacial perturbations.** Same as Fig. 2. Anomalies in (H) are computed relative to *interglacial(permeable sea ice)*.

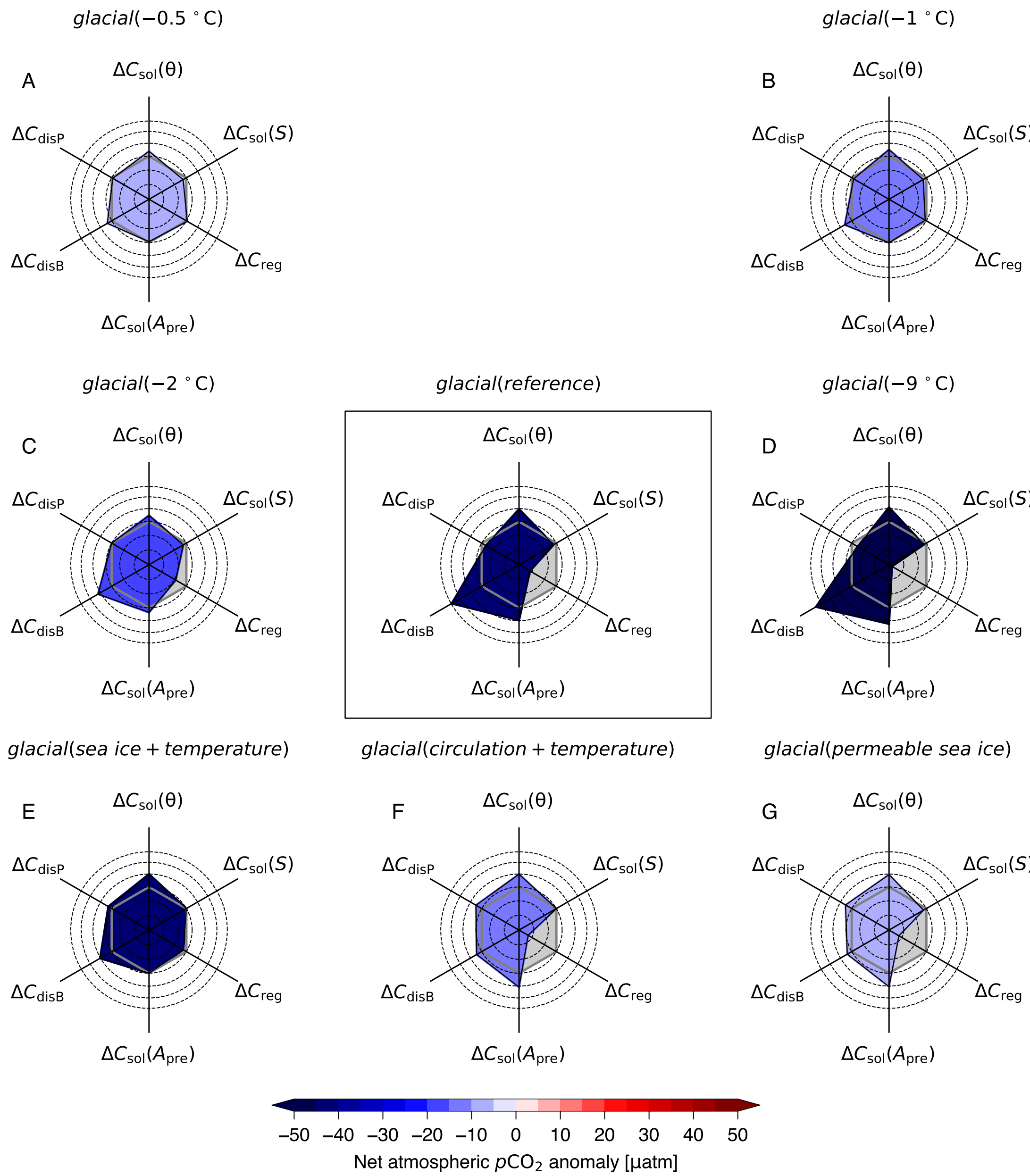

Fig. S9: **Detailed partitioning of oceanic carbon anomalies in response to glacial perturbations.** Same as Fig. 3, but for (A) *glacial(*$-0.5\ ^{\circ}\mathrm{C}$*)*, (B) *glacial(*$-1\ ^{\circ}\mathrm{C}$*)*, (C) *glacial(*$-2\ ^{\circ}\mathrm{C}$*)*, (D) *glacial(*$-9\ ^{\circ}\mathrm{C}$*)*, (E) *glacial(sea ice+temperature)*, (F) *glacial(circulation+temperature)*, and (G) *glacial(permeable sea ice)*. The *glacial(reference)*, positioned between (C) and (D), remains as in Fig. 3A for easier comparison. Oceanic carbon pool and $p\mathrm{CO_2}^{\mathrm{atm}}$ anomalies are computed relative to (A-F) *interglacial(reference)* or (G) *interglacial(permeable sea ice)*.

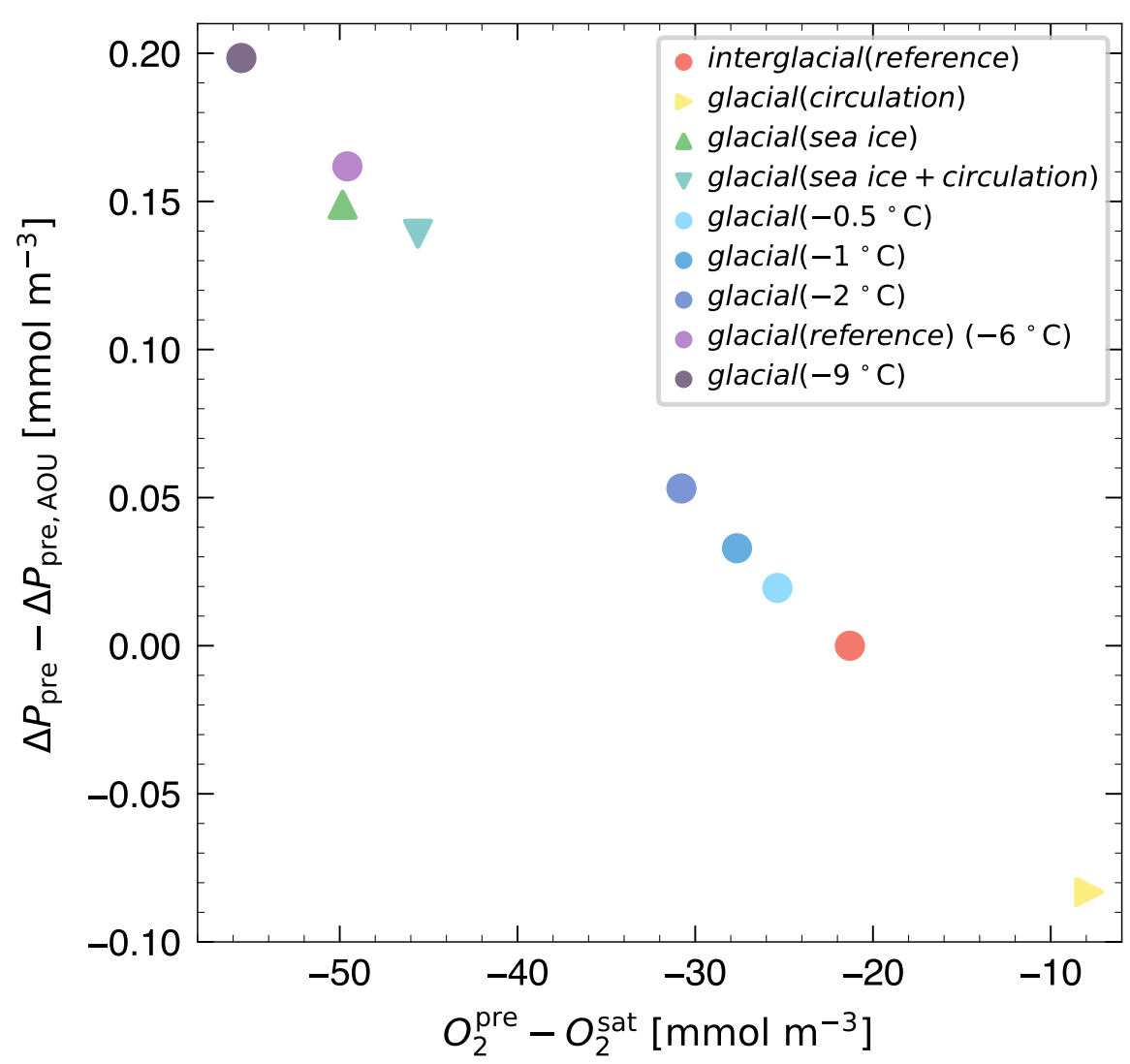

Fig. S10: **Discrepancy between preformed phosphate diagnostics as a function of oxygen undersaturation.** Discrepancy between anomalies of diagnosed preformed phosphate, PO_4^{pre}, and AOU-inferred preformed phosphate, $PO_4^{pre,AOU}$, as a function of oxygen undersaturation, $O_2^{pre} - O_2^{sat}$. Anomalies are relative to the *interglacial(reference)* simulation.

REFERENCES

1. J.-R. Petit, J. Jouzel, D. Raynaud, N. I. Barkov, J.-M. Barnola, I. Basile, M. Bender, J. Chappellaz, M. Davis, G. Delaygue, M. Delmotte, V. M. Kotlyakov, M. Legrand, V. Y. Lipenkov, C. Lorius, L. Pépin, C. Ritz, E. Saltzman, M. Stievenard, Climate and atmospheric history of the past 420,000 years from the Vostok ice core, Antarctica. *Nature* **399**, 429–436 (1999).

2. U. Siegenthaler, T. F. Stocker, E. Monnin, D. Lüthi, J. Schwander, B. Stauffer, D. Raynaud, J.-M. Barnola, H. Fischer, V. Masson-Delmotte, J. Jouzel, Stable carbon cycle–climate relationship during the late Pleistocene. *Science* **310**, 1313–1317 (2005).

3. D. Lüthi, M. Le Floch, B. Bereiter, T. Blunier, J.-M. Barnola, U. Siegenthaler, D. Raynaud, J. Jouzel, H. Fischer, K. Kawamura, T. F. Stocker, High-resolution carbon dioxide concentration record 650,000–800,000 years before present. *Nature* **453**, 379–382 (2008).

4. T. Schneider von Deimling, A. Ganopolski, H. Held, S. Rahmstorf, How cold was the Last Glacial Maximum? *Geophys. Res. Lett.* **33**, L14709 (2006).

5. J. D. Shakun, P. U. Clark, F. He, S. A. Marcott, A. C. Mix, Z. Liu, B. Otto-Bliesner, A. Schmittner, E. Bard, Global warming preceded by increasing carbon dioxide concentrations during the last deglaciation. *Nature* **484**, 49–54 (2012).

6. M. B. Osman, J. E. Tierney, J. Zhu, R. Tardif, G. J. Hakim, J. King, C. J. Poulsen, Globally resolved surface temperatures since the Last Glacial Maximum. *Nature* **599**, 239–244 (2021).

7. C. R. Stokes, L. Tarasov, R. Blomdin, T. M. Cronin, T. G. Fisher, R. Gyllencreutz, C. Hättestrand, J. Heyman, R. C. Hindmarsh, A. L. Hughes, M. Jakobsson, N. Kirchner, S. J. Livingstone, M. Margold, J. B. Murton, R. Noormets, W. R. Peltier, D. M. Peteet, D. J. W. Piper, F. Preusser, H. Renssen, D. H. Roberts, D. M. Roche, F. Saint-Ange, A. P. Stroeven, J. T. Teller, On the reconstruction of palaeo-ice sheets: Recent advances and future challenges. *Quat. Sci. Rev.* **125**, 15–49 (2015).

8. C. L. Batchelor, M. Margold, M. Krapp, D. K. Murton, A. S. Dalton, P. L. Gibbard, C. R. Stokes, J. B. Murton, A. Manica, The configuration of Northern Hemisphere ice sheets through the quaternary. *Nat. Commun.* **10**, 3713 (2019).

9. R. Gersonde, X. Crosta, A. Abelmann, L. Armand, Sea-surface temperature and sea ice distribution of the Southern Ocean at the EPILOG Last Glacial Maximum—A circum-Antarctic view based on siliceous microfossil records. *Quat. Sci. Rev.* **24**, 869–896 (2005).

10. D. M. Roche, X. Crosta, H. Renssen, Evaluating Southern Ocean sea-ice for the Last Glacial Maximum and pre-industrial climates: PMIP-2 models and data evidence. *Quat. Sci. Rev.* **56**, 99–106 (2012).

11. D. M. Sigman, E. A. Boyle, Glacial/interglacial variations in atmospheric carbon dioxide. *Nature* **407**, 859–869 (2000).

12. D. Archer, A. Winguth, D. Lea, N. Mahowald, What caused the glacial/interglacial atmospheric pCO_2 cycles? *Rev. Geophys.* **38**, 159–189 (2000).

13. D. M. Sigman, M. P. Hain, G. H. Haug, The polar ocean and glacial cycles in atmospheric CO_2 concentration. *Nature* **466**, 47–55 (2010).

14. K. E. Kohfeld, A. Ridgwell, "Glacial-interglacial variability in atmospheric CO_2," in *Surface Ocean-Lower Atmosphere Processes*, C. Le Quere, E. S. Saltzman, eds. (American Geophysical Union, 2009), vol. 187, pp. 251–286.

15. E. D. Galbraith, L. C. Skinner, The biological pump during the Last Glacial Maximum. *Ann. Rev. Mar. Sci.* **12**, 559–586 (2020).

16. S. Khatiwala, A. Schmittner, J. Muglia, Air-sea disequilibrium enhances ocean carbon storage during glacial periods. *Sci. Adv.* **5**, eaaw4981 (2019).

17. L. C. Skinner, F. Primeau, E. Freeman, M. de la Fuente, P. A. Goodwin, J. Gottschalk, E. Huang, I. N. McCave, T. L. Noble, A. E. Scrivner, Radiocarbon constraints on the glacial ocean circulation and its impact on atmospheric CO_2. *Nat. Commun.* **8**, 16010 (2017).

18. P. A. Rafter, W. R. Gray, S. K. Hines, A. Burke, K. M. Costa, J. Gottschalk, M. P. Hain, J. W. Rae, J. R. Southon, M. H. Walczak, J. Yu, J. F. Adkins, T. DeVries, Global reorganization of deep-sea circulation and carbon storage after the last ice age. *Sci. Adv.* **8**, eabq5434 (2022).

19. T. Kurahashi-Nakamura, A. Abe-Ouchi, Y. Yamanaka, K. Misumi, Compound effects of Antarctic sea ice on atmospheric pCO_2 change during glacial–interglacial cycle. *Geophys. Res. Lett.* **34**, L20708 (2007).

20. M. P. Hain, D. M. Sigman, G. H. Haug, Carbon dioxide effects of Antarctic stratification, North Atlantic Intermediate Water formation, and subantarctic nutrient drawdown during the last ice age: Diagnosis and synthesis in a geochemical box model. *Global Biogeochem. Cycles* **24**, GB4023 (2010).

21. R. E. Rickaby, H. Elderfield, N. Roberts, C.-D. Hillenbrand, A. Mackensen, Evidence for elevated alkalinity in the glacial Southern Ocean. *Paleoceanography* **25**, PA1209 (2010).

22. V. Brovkin, A. Ganopolski, D. Archer, S. Rahmstorf, Lowering of glacial atmospheric CO_2 in response to changes in oceanic circulation and marine biogeochemistry. *Paleoceanography* **22**, PA4202 (2007).

23. V. Brovkin, A. Ganopolski, D. Archer, G. Munhoven, Glacial CO_2 cycle as a succession of key physical and biogeochemical processes. *Clim. Past.* **8**, 251–264 (2012).

24. T. Ito, M. J. Follows, Preformed phosphate, soft tissue pump and atmospheric CO_2. *J. Mar. Res.* **63**, 813–839 (2005).

25. R. G. Williams, M. J. Follows, *Ocean Dynamics and the Carbon Cycle: Principles and Mechanisms* (Cambridge Univ. Press, 2012).

26. J. M. Lauderdale, A. C. N. Garabato, K. I. Oliver, M. J. Follows, R. G. Williams, Wind-driven changes in Southern Ocean residual circulation, ocean carbon reservoirs and atmospheric CO_2. *Climate Dynam.* **41**, 2145–2164 (2013).

27. J. M. Lauderdale, S. Dutkiewicz, R. G. Williams, M. J. Follows, Quantifying the drivers of ocean-atmosphere CO_2 fluxes. *Global Biogeochem. Cycles* **30**, 983–999 (2016).

28. J. M. Lauderdale, R. G. Williams, D. R. Munday, D. P. Marshall, The impact of Southern Ocean residual upwelling on atmospheric CO_2 on centennial and millennial timescales. *Climate Dynam.* **48**, 1611–1631 (2017).

29. A. Marzocchi, M. F. Jansen, Global cooling linked to increased glacial carbon storage via changes in Antarctic sea ice. *Nat. Geosci.* **12**, 1001–1005 (2019).

30. T. Ito, M. J. Follows, Air-sea disequilibrium of carbon dioxide enhances the biological carbon sequestration in the Southern Ocean. *Global Biogeochem. Cycles* **27**, 1129–1138 (2013).

31. K. Stein, A. Timmermann, E. Y. Kwon, T. Friedrich, Timing and magnitude of Southern Ocean sea ice/carbon cycle feedbacks. *Proc. Natl. Acad. Sci. U.S.A.* **117**, 4498–4504 (2020).

32. J. Marshall, C. Hill, L. Perelman, A. Adcroft, Hydrostatic, quasi-hydrostatic, and nonhydrostatic ocean modeling. *J. Geophys. Res. Oceans* **102**, 5733–5752 (1997).

33. J. Zhang, W. Hibler, M. Steele, D. Rothrock, Arctic ice–ocean modeling with and without climate restoring. *J. Phys. Oceanogr.* **28**, 191–217 (1998).

34. M. Losch, D. Menemenlis, J.-M. Campin, P. Heimbach, C. Hill, On the formulation of sea-ice models. Part 1: Effects of different solver implementations and parameterizations. *Ocean Model.* **33**, 129–144 (2010).

35. S. Dutkiewicz, M. J. Follows, P. Heimbach, J. Marshall, Controls on ocean productivity and air-sea carbon flux: An adjoint model sensitivity study. *Geophys. Res. Lett.* **33**, L02603 (2006).

36. P. Parekh, M. J. Follows, S. Dutkiewicz, T. Ito, Physical and biological regulation of the soft tissue carbon pump. *Paleoceanography* **21**, PA3001 (2006).

37. P. R. Gent, J. McWilliams, Isopycnal mixing in ocean circulation models. *J. Phys. Oceanogr.* **20**, 150–155 (1990).

38. M. Nikurashin, R. Ferrari, Overturning circulation driven by breaking internal waves in the deep ocean. *Geophys. Res. Lett.* **40**, 3133–3137 (2013).

39. P. L. Roe, "Some contributions to the modelling of discontinuous flows," in *Large-Scale Computations in Fluid Mechanics* (American Mathematical Society, 1985), pp. 163–193.

40. L.-P. Nadeau, R. Ferrari, M. F. Jansen, Antarctic sea ice control on the depth of North Atlantic Deep Water. *J. Climate* **32**, 2537–2551 (2019).

41. L.-P. Nadeau, M. F. Jansen, Overturning circulation pathways in a two-basin ocean model. *J. Phys. Oceanogr.* **50**, 2105–2122 (2020).

42. M. F. Jansen, Glacial ocean circulation and stratification explained by reduced atmospheric temperature. *Proc. Natl. Acad. Sci. U.S.A.* **114**, 45–50 (2017).

43. L.-P. Nadeau, M. F. Jansen, S. Gregorio, A simple model for global temperature control on Dansgaard Oeschger oscillations. *J. Climate* **38**, 3169–3184 (2025).

44. C. L. Wolfe, P. Cessi, What sets the strength of the middepth stratification and overturning circulation in eddying ocean models? *J. Phys. Oceanogr.* **40**, 1520–1538 (2010).

45. M. Nikurashin, G. Vallis, A theory of the interhemispheric meridional overturning circulation and associated stratification. *J. Phys. Oceanogr.* **42**, 1652–1667 (2012).

46. A. Martínez-Garcia, A. Rosell-Melé, W. Geibert, R. Gersonde, P. Masqué, V. Gaspari, C. Barbante, Links between iron supply, marine productivity, sea surface temperature, and CO_2 over the last 1.1 Ma. *Paleoceanography* **24**, PA1207 (2009).

47. K. Wallmann, B. Schneider, M. Sarnthein, Effects of eustatic sea-level change, ocean dynamics, and nutrient utilization on atmospheric pCO_2 and seawater composition over the last 130,000 years: A model study. *Clim. Past.* **12**, 339–375 (2016).

48. H. Saini, K. J. Meissner, L. Menviel, K. Kvale, Impact of iron fertilisation on atmospheric CO_2 during the last glaciation. *Clim. Past.* **19**, 1559–1584 (2023).

49. O. Cartapanis, E. D. Galbraith, D. Bianchi, S. L. Jaccard, Carbon burial in deep-sea sediment and implications for oceanic inventories of carbon and alkalinity over the last glacial cycle. *Clim. Past.* **14**, 1819–1850 (2018).

50. M. E. Weber, I. Bailey, S. R. Hemming, Y. M. Martos, B. T. Reilly, T. A. Ronge, S. Brachfeld, T. Williams, M. Raymo, S. T. Belt, L. Smik, H. Vogel, V. L. Peck, L. Armbrecht, A. Cage, F. G. Cardillo, Z. du, G. Fauth, C. J. Fogwill, M. Garcia, M. Garnsworthy, A. Glüder, M. Guitard, M. Gutjahr, I. Hernández-Almeida, F. S. Hoem, J. H. Hwang, M. Iizuka, Y. Kato, B. Kenlee, S. OConnell, L. F. Pérez, O. Seki, L. Stevens, L. Tauxe, S. Tripathi, J. Warnock, X. Zheng, Antiphased dust deposition and productivity in the Antarctic Zone over 1.5 million years. *Nat. Commun.* **13**, 2044 (2022).

51. M. P. Humphreys, E. R. Lewis, J. D. Sharp, D. Pierrot, PyCO2SYS v1. 8: Marine carbonate system calculations in Python. *Geosci. Model Dev.* **15**, 15–43 (2022).

52. M. P. Humphreys, A. J. Schiller, D. E. Sandborn, L. Gregor, D. Pierrot, S. M. A. C. van Heuven, E. R. Lewis, D. W. R. Wallace, PyCO2SYS: Marine carbonate system calculations in Python, Zenodo (2022); https://zenodo.org/records/6560756.

53. T. Ito, M. Follows, E. Boyle, Is AOU a good measure of respiration in the oceans? *Geophys. Res. Lett.* **31**, L17305 (2004).

54. L. D. Talley, Closure of the global overturning circulation through the Indian, Pacific, and Southern Oceans: Schematics and transports. *Oceanography* **26**, 80–97 (2013).

55. J. Muglia, A. Schmittner, Carbon isotope constraints on glacial Atlantic meridional overturning: Strength vs depth. *Quat. Sci. Rev.* **257**, 106844 (2021).

56. S. K. Lauvset, N. Lange, T. Tanhua, H. C. Bittig, A. Olsen, A. Kozyr, S. Alin, M. Álvarez, K. Azetsu-Scott, L. Barbero, S. Becker, P. J. Brown, B. R. Carter, L. C. da Cunha, R. A. Feely, M. Hoppema, M. P. Humphreys, M. Ishii, E. Jeansson, L.-Q. Jiang, S. D. Jones, C. Lo Monaco, A. Murata, J. D. Müller, F. F. Pérez, B. Pfeil, C. Schirnick, R. Steinfeldt, T. Suzuki, B. Tilbrook, A. Ulfsbo, A. Velo, R. J. Woosley, R. M. Key, GLODAPv2.2022: The latest version of the global interior ocean biogeochemical data product. *Earth Syst. Sci. Data* **14**, 5543–5572 (2022).

57. X. Sun, K. Matsumoto, Effects of sea ice on atmospheric pCO_2: A revised view and implications for glacial and future climates. *J. Geophys. Res. Biogeosci.* **115**, G02015 (2010).

58. M. Gupta, M. J. Follows, J. M. Lauderdale, The effect of Antarctic sea ice on Southern Ocean carbon outgassing: Capping versus light attenuation. *Global Biogeochem. Cycles* **34**, e2019GB006489 (2020).

59. W. B. Curry, D. W. Oppo, Glacial water mass geometry and the distribution of $\delta^{13}C$ of ΣCO_2 in the western Atlantic Ocean. *Paleoceanography* **20**, PA1017 (2005).

60. R. Ferrari, M. F. Jansen, J. F. Adkins, A. Burke, A. L. Stewart, A. F. Thompson, Antarctic sea ice control on ocean circulation in present and glacial climates. *Proc. Natl. Acad. Sci. U.S.A.* **111**, 8753–8758 (2014).

61. J. L. Sarmiento, N. Gruber, M. Brzezinski, J. Dunne, High-latitude controls of thermocline nutrients and low latitude biological productivity. *Nature* **427**, 56–60 (2004).

62. J. Palter, J. L. Sarmiento, A. Gnanadesikan, J. Simeon, R. Slater, Fueling export production: Nutrient return pathways from the deep ocean and their dependence on the Meridional Overturning Circulation. *Biogeosciences* **7**, 3549–3568 (2010).

63. F. Fripiat, A. Martínez-García, D. Marconi, S. E. Fawcett, S. H. Kopf, V. H. Luu, P. A. Rafter, R. Zhang, D. M. Sigman, G. H. Haug, Nitrogen isotopic constraints on nutrient transport to the upper ocean. *Nat. Geosci.* **14**, 855–861 (2021).

64. R. Wanninkhof, Relationship between wind speed and gas exchange over the ocean. *J. Geophys. Res. Oceans* **97**, 7373–7382 (1992).

65. I. Marinov, A. Gnanadesikan, J. L. Sarmiento, J. Toggweiler, M. Follows, B. Mignone, Impact of oceanic circulation on biological carbon storage in the ocean and atmospheric pCO_2. *Global Biogeochem. Cycles* **22**, GB3007 (2008).

66. T. Vollmer, T. Ito, J. Lynch-Stieglitz, Proxy-based preformed phosphate estimates point to increased biological pump efficiency as primary cause of Last Glacial Maximum CO_2 drawdown. *Paleoceanogr. Paleoclimatol.* **37**, e2021PA004339 (2022).

67. M. Nikurashin, R. Ferrari, Global energy conversion rate from geostrophic flows into internal lee waves in the deep ocean. *Geophys. Res. Lett.* **38**, L08610 (2011).

68. D. Lund, J. Adkins, R. Ferrari, Abyssal atlantic circulation during the Last Glacial Maximum: Constraining the ratio between transport and vertical mixing. *Paleoceanography* **26**, PA1213 (2011).

69. J. Yu, L. Menviel, Z. Jin, D. Thornalley, S. Barker, G. Marino, E. Rohling, Y. Cai, F. Zhang, X. Wang, Y. Dai, P. Chen, W. S. Broecker, Sequestration of carbon in the deep Atlantic during the last glaciation. *Nat. Geosci.* **9**, 319–324 (2016).

70. L. Menviel, J. Yu, F. Joos, A. Mouchet, K. Meissner, M. H. England, Poorly ventilated deep ocean at the Last Glacial Maximum inferred from carbon isotopes: A data-model comparison study. *Paleoceanography* **32**, 2–17 (2017).

71. J. Muglia, L. C. Skinner, A. Schmittner, Weak overturning circulation and high Southern Ocean nutrient utilization maximized glacial ocean carbon. *Earth Planet. Sci. Lett.* **496**, 47–56 (2018).

72. H. Kobayashi, A. Oka, A. Yamamoto, A. Abe-Ouchi, Glacial carbon cycle changes by Southern Ocean processes with sedimentary amplification. *Sci. Adv.* **7**, eabg7723 (2021).

73. A. Schmittner, N. Fillman, Carbon and carbon-13 in the preindustrial and glacial ocean. *PLOS Clim.* **3**, e0000434 (2024).

74. A. W. Omta, C. Follett, J. Lauderdale, R. Ferrari, Carbon isotope budget indicates biological disequilibrium dominated ocean carbon storage at the Last Glacial Maximum. *Nat. Commun.* **15**, 8006 (2024).

75. W. R. Gray, M. Trudgill, M. Shankle, S. Eggleston, C. de Lavergne, E. D. Galbraith, Disequilibrium and soft tissue pump contributions to glacial CO_2 drawdown. *AGU Advances* **7**, e2025AV002016 (2026).

76. X. T. Wang, D. M. Sigman, M. G. Prokopenko, J. F. Adkins, L. F. Robinson, S. K. Hines, J. Chai, A. S. Studer, A. Martínez-García, T. Chen, G. H. Haug, Deep-sea coral evidence for lower Southern Ocean surface nitrate concentrations during the last ice age. *Proc. Natl. Acad. Sci. U.S.A.* **114**, 3352–3357 (2017). https://doi.org/10.1073/pnas.1615718114.

77. J. Marshall, T. Radko, A model of the upper branch of the meridional overturning of the southern ocean. *Progr. Oceanogr.* **70**, 331–345 (2006).

78. R. A. Green, L. Menviel, K. J. Meissner, X. Crosta, D. Chandan, G. Lohmann, W. R. Peltier, X. Shi, J. Zhu, Evaluating seasonal sea-ice cover over the Southern Ocean at the Last Glacial Maximum. *Clim. Past.* **18**, 845–862 (2022).

79. S. Levitus, T. P. Boyer, "World ocean atlas 1994. Vol. 4, temperature" (Tech. Rep., National Environmental Satellite, Data, and Information Service, 1994).

80. B. Bereiter, S. Shackleton, D. Baggenstos, K. Kawamura, J. Severinghaus, Mean global ocean temperatures during the last glacial transition. *Nature* **553**, 39–44 (2018).

81. MARGO Project Members, Constraints on the magnitude and patterns of ocean cooling at the Last Glacial Maximum. *Nat. Geosci.* **2**, 127–132 (2009).

82. C. L. Parkinson, Global sea ice coverage from satellite data: Annual cycle and 35-yr trends. *J. Climate* **27**, 9377–9382 (2014).

83. R. Lumpkin, K. Speer, Global ocean meridional overturning. *J. Phys. Oceanogr.* **37**, 2550–2562 (2007).

84. D. R. Jackett, T. J. Mcdougall, Minimal adjustment of hydrographic profiles to achieve static stability. *J. Atmos. Oceanic Tech.* **12**, 381–389 (1995).

85. W. G. Large, S. G. Yeager, *Diurnal to Decadal Global Forcing for Ocean and Sea-Ice Models: The Data Sets and Flux Climatologies*" (Climate and Global Dynamics Division, National Center for Atmospheric Research, 2004).

86. L. A. Anderson, J. L. Sarmiento, Redfield ratios of remineralization determined by nutrient data analysis. *Global Biogeochem. Cycles* **8**, 65–80 (1994).

87. R. Weiss, Carbon dioxide in water and seawater: The solubility of a non-ideal gas. *Mar. Chem.* **2**, 203–215 (1974).

88. P. Goodwin, M. J. Follows, R. G. Williams, Analytical relationships between atmospheric carbon dioxide, carbon emissions, and ocean processes. *Global Biogeochem. Cycles* **22**, GB3030 (2008).

89. M. Ödalen, J. Nycander, K. I. Oliver, L. Brodeau, A. Ridgwell, The influence of the ocean circulation state on ocean carbon storage and CO_2 drawdown potential in an Earth system model. *Biogeosciences* **15**, 1367–1393 (2018).

90. W. S. Broecker, "NO", a conservative water-mass tracer. *Earth Planet. Sci. Lett.* **23**, 100–107 (1974).

91. R. Bernardello, I. Marinov, J. B. Palter, J. L. Sarmiento, E. D. Galbraith, R. D. Slater, Response of the ocean natural carbon storage to projected twenty-first-century climate change. *J. Climate* **27**, 2033–2053 (2014).
